# Parameter estimation in systems biology models using spline approximation

**DOI:** 10.1186/1752-0509-5-14

**Published:** 2011-01-24

**Authors:** Choujun Zhan, Lam F Yeung

**Affiliations:** 1 Department of Electronic Engineering, City University of Hong Kong, Hong Kong, PR China

## Abstract

**Background:**

Mathematical models for revealing the dynamics and interactions properties of biological systems play an important role in computational systems biology. The inference of model parameter values from time-course data can be considered as a "reverse engineering" process and is still one of the most challenging tasks. Many parameter estimation methods have been developed but none of these methods is effective for all cases and can overwhelm all other approaches. Instead, various methods have their advantages and disadvantages. It is worth to develop parameter estimation methods which are robust against noise, efficient in computation and flexible enough to meet different constraints.

**Results:**

Two parameter estimation methods of combining spline theory with Linear Programming (LP) and Nonlinear Programming (NLP) are developed. These methods remove the need for ODE solvers during the identification process. Our analysis shows that the augmented cost function surfaces used in the two proposed methods are smoother; which can ease the optima searching process and hence enhance the robustness and speed of the search algorithm. Moreover, the cores of our algorithms are LP and NLP based, which are flexible and consequently additional constraints can be embedded/removed easily. Eight system biology models are used for testing the proposed approaches. Our results confirm that the proposed methods are both efficient and robust.

**Conclusions:**

The proposed approaches have general application to identify unknown parameter values of a wide range of systems biology models.

## Background

In recent years, the rapid development of sophisticated experiment tools in molecular biology allows the acquisition of high qualitative time series data which can significantly improve the ability of revealing the complex dynamics and interactions of biological systems. Profiting from the rapid technological advances, more and more researchers from different disciplines can now utilize such observation data to establish mechanism-based models which can incorporate every possible detail and functioning of biological systems [1]. One common approach is to characterize the biological system with a set of Ordinary Differential Equations (ODEs) [2-7]. Generally, there are two major aspects of building an ODE model for a biological system from experimentally measured time series: (1) to determine the structure of the system through a set of suitable ODEs with unknown parameters; (2) to determine the unknown parameters of this ODE model. The identification of these unknown parameter with fixed model structure from observations is one of the central issues of computational systems biology [8]. This type of approach can be considered as a "reverse engineering process" [9-11].

The parameter estimation problem is generally formulated as an optimization problem that minimizes an objective function which represents the fitness of the model with respect to a set of experimental data [8,12-17]. Two major optimization approaches are commonly adopted; the gradient-based nonlinear optimization method and the evolutionary based method. Also, simulations had shown that the simulated annealing (SA) method can offer promising results [18]. In [19], many deterministic and stochastic global optimization (GO) methods for parameter estimation were further compared using a three-step pathway model with noise free data assumption; the best result was given by the Stochastic Ranking Evolution Strategy (SRES) method. It is worth mentioning that, due to its simplicity in implementation, evolutionary algorithms, such as genetic algorithm and their variants, are extensively utilized for identifying unknown parameters of systems biology models [1,11,20-22]. However, most of these aforementioned approaches need a numerical ODE solver to perform the numerical integration for the underlining differential equations. Studies have revealed that more than 90% of the computation time is consumed in the ODE solver during the identification process [19]. In particular, for nonlinear dynamical systems with high-parameter-dimension, one trial usually consumes tens of hours or even days [10,20,21,23]. Furthermore, the convergence property is aggravated by numerical integration failure, which is a major problem in the optimization process [11]. The computational burden can be relieved by reformulating the system involving differential equations into a system of algebraic equations [12,15,17,24], which can be classified as "decomposition approaches". These decomposition approaches are widely employed in the parameter estimation of S-systems [7,25]. The reliability of the decomposed methods depends on the accuracy of the "smooth" estimated derivatives and the states of the system. In practice, these data are subject to significant observation noise. Without proper pre-processing, the estimation faces the potential of the overfitting problem and hence the estimation can deviate badly from the "true" value [26,27]. Regularization can be considered as a mathematical pre-processing on the measured noisy data set and be used to control the trade-off between the "roughness" of the solution and the infidelity of the data [28]. Since we are dealing with a known structured bio-system, the system model itself possesses a physical inertia and can serve as physical constraints which limit the system states within a set of possible trajectories. In this paper, the over-fitting problem can be relieved by embedding the model dynamics, the mass and energy balance constraints into our constrained optimization algorithms. Owing to the nonlinearity of systems biology models, the cost function to be minimized is complex and has multiple local minima. Minimization algorithms face the high possibility of getting trapped at local optima. For these reasons, the parameter estimation problem is still a bottleneck and a challenging task of computational analysis of systems biology [1,11]. Until now, none of the parameter estimation methods is effective in all cases and can overwhelm all the other methods. Instead, various methods have their advantages and disadvantages. Consequently, it is worthy to develop acceptably "good enough" methods within a given tolerance and time frame.

For practical purpose, some essential issues should be taken into account when developing a parameter estimation method: first, the method must be "efficient" enough that a trial can be completed within a reasonable computation time; second, for biological systems, the observation data is often corrupted by high level of noise, which complicates the objective function surface and introduces unwanted additional local minima in the search space [29]. Hence, the approach should be robust subject to noise; third, it needs to be flexible enough for adding/removing physical constraints, such as model dynamics, the mass and energy balance constraints. Furthermore, the representative cost function should have less local minima so as to ease the optimization algorithm in converging to the global minima. In this paper, two parameter estimation methods of combining spline theory [28] with Linear Programming (LP) and Nonlinear Programming (NLP) are developed, respectively. These methods remove the need for an ODE solver. Our analysis exhibits that the cost function surfaces of the two proposed methods are smooth.

Moreover, the cores of our algorithms are LP and NLP based, which are very flexible and hence additional constraints can be embeded/removed easily. Eight systems biology models were used to test the proposed algorithms. Experimental results show that the proposed methods are both efficient and robust (see additional file 1 for details).

This paper is organized as follows: The preliminary problem formulation is given and the bottleneck of the problem is highlighted in the next section. Then, two parameter estimation methods surmounting those bottlenecks are presented. In section 3, two trials are given, a simple enzyme kinetic model and the mammalian G1/S transition network model, in order to illustrate the robustness and the effectiveness of these two proposed methods (more models and trial results are given in additional file 1 and 2). Finally, conclusions and discussions are given in section 4.

## Methods

### Parameter estimation problem of systems biology models

Biological pathway dynamics can be modelled by the following continuous ODEs:

(1) $$\begin{array}{l} \dot{x}(t)=f(x(t),u(t),\theta ),\;\;\;x(t_{0})=x_{0},\\ y(t)=g(x(t))+\eta (t), \end{array}$$

where *x *ϵ *R^n ^*is the system's state vector (for example the concentrations of a process), *θ *ϵ *R^k ^*is the system's parameter vector (for instance, the reaction rates), *u*(*t*) ϵ *R^p ^*is system's input, *y *ϵ *R^m ^*denotes the measured data subject to a Gaussian white noise *η*(*t*) ~ *N*(0, σ^2^), and *x*_0 _is the initial state. *f*(·) is a set of nonlinear transition functions describing the dynamical properties of a biological system. Here, *g*(·) represents a measurement function. If all the states can be measured, the observer *g*(·) becomes an identity matrix. Otherwise, *g*(·) usually is a rectangular zero-one matrix with corresponding rows deleted (represent the immeasurable states) from the identity matrix *I_n_*.

The parameter estimation problem of nonlinear dynamical systems described in (1) can be formulated as a nonlinear programming problem (NLP) P_0 _with differential-algebraic constraints:

(2) $$\begin{array}{l} {\text{P}}_{0}:\underset{\hat{\theta },{\hat{x}}_{0}}{\min}\sum_{j=0}^{N-1}\sum_{i=1}^{n}w_{ij}{\left\|y_{i}(t_{j})-{\hat{y}}_{i}(t_{j}|\hat{\theta })\right\|}_{l},\\ s.t.\;\{\begin{array}{ll} \left(\text{i}\right) & \hat{\dot{x}}(t_{j})=f(\hat{x}(t_{j}|\hat{\theta }),u(t),\hat{\theta }),\;x(t_{0})={\hat{x}}_{0},\\ \left(\text{ii}\right) & \hat{y}(t_{j})=g(\hat{x}(t_{j}|\hat{\theta })),\;\;j=1,2,\cdots ,N-1,\\ \left(\text{iii}\right) & C_{eq}(\hat{x},\hat{\dot{x}},\hat{\theta })=0,\\ \left(\text{iv}\right) & c_{ineq}(\hat{x},\hat{\dot{x}},\hat{\theta })\leq 0,\\ \left(\text{v}\right) & \theta_{L}\leq \hat{\theta }\leq \theta_{U}. \end{array} \end{array}$$

P_0 _minimizes a cost function that measures the fitness of the model with respect to a given set of experiment data subjecting to a set of constraints, where $\hat{\theta }\in R^{k}$ is the set of parameters to be estimated, ||·||*_l _*denotes the *l*-norm with *l *> 0, ${\hat{x}}_{0}$ is the estimated initial condition, $\hat{x}\in R^{k}$ is the estimated system states ($\hat{x}(t_{j}|\hat{\theta })$ represents the estimated variable at time *t_j _*with parameter $\hat{\theta }$ and initial condition ${\hat{x}}_{0}$), *w_ij _*are the weighting coefficients, $\hat{y}$ is the estimated measured data. In some applications, additional constraints are introduced to impose special structural properties of a given system; they can be implemented in the form of the equality and inequality constraints *C_eq _*and *C_ineq _*(for instance the system performance and the mass balance constraints). Finally, *θ *_L _and *θ *_U _are simple structural constraints such as the parameter's upper/lower bounds (they can be part of the *C_ineq_*).

For the NLP-P_0 _, the direct optimization methods, such as Newton type methods and many GO methods, require solving the nonlinear dynamic model (1) for $\hat{x}$ in order to compute the cost function. The common method to estimate $\hat{\dot{x}}(t_{i})$ and $\hat{x}(t_{i})$ is using ODE solvers, which perform the numerical integration with $\hat{\theta }$ fixed at each iteration [19]. During the process of identification, the integration has to be executed thousands, even millions of times. That is the main reason more than 90% of the time is consumed in the ODE solver [24] and the computation time spent on the P_0 _can be hours even days [10,20]. Moreover, P_0 _is a nonlinear optimization problem subjecting to a set of linear and non-linear differential equation constraints. Hence, P_0 _is often multimodal (non-convex) and has many local minima. In a high-noise environment, the situation becomes more difficult. Consequently, P_0 _requires further manipulation in order to reduce the complexity so as to relieve the computation burden and also to avoid being trapped in local minima.

Instead of using ODE solvers to estimate *x*(*t*) and $\dot{x}(t)$, one can utilize spline approximation. Given *L *real values *τ_i_*, called knots, with *τ*_0 _≤ τ_1 _*≤ *· · · ≤ τ_*L*-1_. Using the Cox-de Boor recursion formula, the B-spline basis of degree *n*_*d *_= 0, 1, 2, · · ·, *L *- 2 can be defined as follows:

(3) $$b_{m,0}(t)≜\{\begin{array}{cccc} 1 & \text{if} & \tau_{m}\leq t<\tau_{m+1}, & m=0,1,\cdots ,L-2,\\ 0 & & \text{otherwise,} & \end{array}$$

(4) $$\begin{array}{l} b_{m,n_{d}}(t)≜\frac{t-\tau_{m}}{\tau_{m+n_{d}}-\tau_{m}}b_{m,n_{d}-1}(t)+\frac{\tau_{m+n_{d}+1}-t}{\tau_{m+n_{d}+1}-\tau_{m+1}}b_{m+1,n_{d}-1}(t),\\ \;\;\;m=0,1,\;\cdots ,\;L-n_{d}-2. \end{array}$$

Let $b_{i}(t)={\left[b_{1,n_{d}}(t),b_{2,n_{d}}(t),\cdots ,b_{L_{i}-n_{d}-2,n_{d}}(t)\right]}^{T}$, a vector of length *L_i _*- *n_d _*- 2, be the chosen basis functions. Then, the estimated variable $\hat{x}$ can be expressed in terms of the basis function expansion [28]

(5) $${\hat{x}}_{i}(t)=\sum_{m=1}^{L_{i}-n_{b}-2}p_{i,m}\cdot b_{i,n_{b}}(t),\;\;\;t\in [t_{0}\;\;\;t_{N}]$$

where ${\hat{x}}_{i}\in R$ is the estimation of the *i*th state of (1), *p*_*i*, *m *_is the weighting coefficient. Let $p_{i}={\left[p_{i,0},p_{i,1},\cdots ,p_{i,L_{j}-n_{d}-2}\right]}^{T}$, (5) can be rewritten in matrix form

(6) $${\hat{x}}_{i}(t)=b_{i}^{T}(t)\cdot p_{i},\;\;\;\;i=1,2,\;\cdots ,\;n.$$

Similarly, the estimated ${\hat{\dot{x}}}_{i}(t)$ can be approximated by

(7) $${\hat{\dot{x}}}_{i}(t)={\dot{b}}_{i}^{T}(t)\cdot p_{i},$$

where ${\dot{b}}_{j}(t)={\left[{\dot{b}}_{0,n_{d}}(t),{\dot{b}}_{0,n_{d}}(t),\cdots ,{\dot{b}}_{L_{j}-n_{d}-2,n_{d}}(t)\right]}^{T}$ is the set of the derivatives of the basis functions. There are various types of splines suitable for this application, such as cubic spline, B-spline, uniform spline, nonuniform spline and interpolating spline. For more detail information about spline approximation theory, please refer to chapter (IX, XI, and XIV) in [28]. As B-spline is simple in formation and efficient for computation, it is adopted here. Our extensive tests have shown that uniform B-spline basis with $\frac{N}{3}\leq L_{i}\leq \frac{N}{2}$ produces good results. Hence, in this paper, unless otherwise indicated, the uniform B-spline basis with $L_{i}\approx \frac{N}{3}$ was used in the parameter identification process.

Next, two techniques based on spline for parameter estimation will be proposed: one is based on linear programming (LP) which is very efficient and can cover many special structured systems and the other one is based on NLP which is flexible and can cater for general system structures.

### The LP Approach

In many bio-system models, *f *(*x*, *θ*) is autonomous system and linear in *θ *as follows:

(8) $$\dot{x}(t)=\Phi (x(t))\theta ,\;\;x(t_{0})=x_{0}.$$

where Φ(*x*) ϵ *R*^*n *×*k *^is a matrix and its elements are a function of the state *x*. Systems with structure (8) covers a large set of systems biology models, such as enzyme kinetic pathway model, RKIP pathway model, I*κ*B-NF-*κ*B model TNF*α*-Mediated NF-*κ*B-signaling pathway model, irreversible inhibition of HIV proteinase model, Laub and Loomis model [2-4,30]. In addition, these types of models are usually subject to the mass balance constraints which can be incorporated into the LP easily (It is demonstrated in the results section via the Enzyme kinetic model).

For noisy data, good smoothing approximation can be achieved by minimizing the following cost function [26]

(9) $$C_{i}=\frac{1}{N+1}\sum_{j=0}^{N}{\left(y_{i}(t_{j})-{\hat{x}}_{i}(t_{j})\right)}^{2}+\lambda \sum_{j=0}^{N}{\left({\hat{x}}_{i}^{\left(m\right)}(t_{j})\right)}^{2}$$

where ${\hat{x}}_{i}^{\left(m\right)}$ represents the *m*th derivative of ${\hat{x}}_{i}$, *m *ϵ *Z*^+ ^and λ ≥ 0 control the trade-off between the "roughness" of the solution and hence can be used to relieve the overfitting problem. If the equality *C_eq _*represents the mass balance constraint and the inequality constraint *C_ineq _*represents parameter values' lower/upper bounds, the B-coefficient vector *p *= [*p*_1_; *p*_2_; · ·· *p_n _*]^*T *^can be computed by solving the following quadratic programming sub-problem A_1 _:

(10) $$\begin{array}{l} {\text{A}}_{1}:\;\underset{p}{\min}\left\{\frac{1}{N+1}\sum_{j=0}^{N}||y(t_{j})-\hat{x}(t_{j})|{|}_{2}^{2}+\lambda \sum_{j=0}^{N}||{\hat{x}}^{\left(m\right)}(t_{j})|{|}_{2}^{2}\right\}\\ s.t.\;\{\begin{array}{ll} \left(\text{i}\right) & A_{eq}\cdot \hat{x}=b_{eq},\\ \left(\text{ii}\right) & {\hat{x}}_{i}(t_{j})=b_{i}^{T}(t_{j})\cdot p_{i},\\ \left(\text{iii}\right) & {\hat{x}}_{i}^{\left(m\right)}(t_{j})={\left(b_{i}^{\left(m\right)}\right)}^{T}(t_{j})\cdot p_{i},\\ & i,=1,2,\cdots n,j=0,1,2\cdots N. \end{array} \end{array}$$

$A_{eq}\cdot \hat{x}=b_{eq}$ stands for the equality constraints $C_{eq}(\hat{x},\hat{\theta })=0$. *A_eq _*is a constant matrix, *b_eq _*is a constant vector. It is found empirically that *m *= 2 and 0 ≤ *λ *≤ 0.05 produce relatively good results. Hence, the parameters *m *= 2, and *λ *= {0, 0.01, 0.03} corresponding to a noise level {0%, 5%, 10%}, respectively, were used in this study. Then, the "smooth" estimated state $\hat{x}$ can be generated by the B-spline approximation (5).

Replace *x*(*t*) by the estimated state $\hat{x}(t)$ and integrate (8) yield:

(11) $$\tilde{x}(t_{j})=\left(\int_{t=t_{0}}^{t_{j}}\Phi (\hat{x}(t))dt\right)\;\;\cdot \;\;\hat{\theta }+{\hat{x}}_{0}={\hat{\Psi }}_{j}\hat{\theta }+{\hat{x}}_{0}.$$

where ${\hat{\Psi }}_{j}=\int_{t=t_{0}}^{t_{j}}\Phi (\hat{x}(t))dt$ represents the transition matrix. Then, P_0 _can be reformulated into the following optimization problem:

(12) $$\begin{array}{l} P_{1}:\underset{\hat{\theta }\in R^{k}}{\min}\sum_{j=0}^{N}\sum_{i=1}^{n}w_{ij}|y_{i}(t_{j})-{\tilde{x}}_{i}(t_{j})|\\ s.t.\;\{\begin{array}{ll} \left(\text{i}\right) & \tilde{x}(\hat{\theta },t_{j})-{\hat{\Psi }}_{j}\hat{\theta }+{\hat{x}}_{0},\\ \left(\text{ii}\right) & \theta_{L}\leq \hat{\theta }\leq \theta_{U},\\ & i=1,..,n;\;j=0,1,2,\cdots ,N. \end{array} \end{array}$$

Here, *w_ij _*≥ 0 is weighting factor. Note that the *L*_1_-norm of a variable × is equivalent to the following relation: $\left|x|=\underset{\alpha \geq 0}{\min}\{\alpha \right\}$; *s.t*. - *α *≤ *x *≤ *α*. Then P_1 _can be transformed into the following augmented optimization problem by introducing the slack variables *α *as follows:

(13) $$\begin{array}{l} {\text{P}}_{2}:\underset{\hat{\theta },\alpha }{\min}\left\{\sum_{i,j}w_{ij}\cdot \alpha_{ij}\right\}\\ s.t.\;\{\begin{array}{ll} \left(\text{i}\right) & -\alpha_{ij}\leq y_{i}(\hat{\theta },t_{j})-{\tilde{x}}_{i}(t_{j}),\\ \left(\text{ii}\right) & \alpha_{ij}\geq y_{i}(\hat{\theta },t_{j})-{\tilde{x}}_{i}(t_{j}),\\ \left(\text{iii}\right) & \tilde{x}(\theta ,t_{j})={\hat{\Psi }}_{j}\hat{\theta }+{\hat{x}}_{0}\\ \left(\text{iv}\right) & \alpha_{ij}\geq 0.\\ \left(\text{v}\right) & \theta_{L}\leq \hat{\theta }\leq \theta_{U},\\ & i=1,..,n;\;j=0,1,...,N. \end{array} \end{array}$$

It is a Linear Programming (LP) problem with variable {*α*, *θ*}, which is a convex problem with a wealth of fast and efficient routines available [31,32].

### Combine spline theory and NLP

To deal with systems biology models, of which the states and parameters are separable, the LP approach is suitable and efficient. In contrast, if the model does not belong to this category, such as the mammalian G1/S transition model and S-system model, the aforementioned approach cannot apply. Thus, a more general approach will be introduced. Recalling (6) and (7), the estimation of $\hat{x}(t_{j})$ and $\hat{\dot{x}}(t_{j})$ can be constructed by a set of basis functions. We can replace $\hat{x}(t_{j})$ and $\hat{\dot{x}}(t_{j})$ in (i-iv) of P_0 _with (6) and (7). With little change, P_0 _can be reformulated as

(14) $$\begin{array}{l} {\text{P}}_{3}:\underset{\hat{\theta },p}{\min}\sum_{j=0}^{N}{\left(y(t_{j})-\hat{y}(t_{j}|\hat{\theta })\right)}^{T}w_{j}(y(t_{j})-\hat{y}(t_{j}|\hat{\theta }))\\ s.t.\;\{\begin{array}{ll} \left(\text{i}\right) & {\hat{x}}_{i}(t_{j})=b_{i}^{T}(t_{j})\cdot p_{i},\\ \left(\text{ii}\right) & {\hat{\dot{x}}}_{i}(t_{j})=b_{i}^{T}(t_{j})\cdot p_{i},\\ \left(\text{iii}\right) & {\left\|\hat{\dot{x}}(t_{j})-f(\hat{x}(t_{j}),u(t),\hat{\theta })\right\|}_{2}^{2}=0,\\ \left(\text{iv}\right) & \hat{y}(t_{i})=g(\hat{x}(t_{j}))\\ \left(\text{v}\right) & C_{eq}(\hat{x}(t_{j}),\hat{\dot{x}}(t_{j}),\hat{\theta })=0,\\ \left(\text{vi}\right) & C_{ineq}(\hat{x}(t_{j}),\hat{\dot{x}}(t_{j}),\hat{\theta })\leq 0,\\ \left(\text{vii}\right) & \theta_{L}\leq \hat{\theta }\leq \theta_{U},\\ & i=1,2,\cdots ,n,\;j=0,1,\cdots ,N. \end{array} \end{array}$$

Note that the constraint-(i) of (2) has been replaced by constraints (i)-(iii) of P_3_. Then, NLP-P_0_-(2) with differential-algebraic constraints turns into NLP-P_3_-(14) with only algebraic equation constraints. Hence, P_3 _does not require ODE solvers, which eases the computation burden (as shown in Examples). In contrast to the the decomposition methods [12,15], which divide the estimation of the system states (and its derivative) and the parameter estimation into two separate steps, P_3 _computes the estimated states (and its derivative) and parameter values at the same time. Note that constraint (iii) of P_3 _governs the estimated state (and its derivative) so as to ensure these estimates belong to the trajectory $\hat{x}(t|\hat{\theta })$, which is a solution of system (1). Thus, the system model itself serves as a filter performing regularization. Hence, the overfitting problem can be relieved (see additional file 2 for details).

For a non-linear system, the Lagrangian of (2), $L(\hat{\theta },{\hat{X}}_{0})$, is an implicit function of $\left\{\hat{\theta },{\hat{x}}_{0}\right\}$[31]. However, many traditional optimization algorithms require the derivative $\partial L/\partial \hat{\theta }$ during the optimization process. As $L(\hat{\theta },{\hat{x}}_{0})$ is an implicit function of $\hat{\theta }$, $\partial L/\partial \hat{\theta }$ cannot be obtained directly, but has to be computed via approximation methods [16], which makes the algorithm unreliable. For P_3_, the Lagrangian $L(\hat{\theta },p)$ now consists of simple algebraic constraints. Thus, $\partial L/\partial \hat{\theta }$ and *∂L*/*∂p *are explicit functions of $\hat{\theta }$ and *p*. In conclusion, many of the aforementioned difficulties can be reduced. P_3 _can be solved by a number of optimization approaches; either via evolution type algorithms, such as genetic algorithm (GA), simulated annealing (SA) and etc, or via traditional NLP algorithms, such as sequential quadratic programming(SQP), sequential penalty function, the trust region approach and etc [33,34].

## Results

Two biological system models, a simple enzyme kinetic model and the mammalian G1/S transition network model, are chosen as benchmarks for evaluating the performance of P_2 _and P_3 _respectively.

### Enzyme kinetic model

Consider the well-known simplified enzyme kinetic model. *E *is the concentration of an enzyme that combines with a substrate *S *to form an enzyme-substrate complex *ES *with a rate constant *k*_1_. The complex *ES *holds two possible out comes in the next step. It can be dissociated into *E *and *S *with a rate constant *k*_2_, or it can further proceed to form a product *P *with a rate constant *k*_3_. It is assumed that none of the products reverts to the initial substrate. These relations can be represented by the following set of ODEs.

(15) $$\begin{array}{l} \frac{dS(t)}{dt}=-k_{1}\cdot E(t)\cdot S(t)+k_{2}\cdot ES(t),\\ \frac{dE(t)}{dt}=-k_{1}\cdot E(t)\cdot S(t)+(k_{2}+k_{3}).\;ES\;(t),\\ \frac{dES(t)}{dt}=k_{1}\cdot \;E\;(t)\cdot S(t)-(k_{2}+k_{3})\;\cdot ES\;(t)\\ \frac{dP(t)}{dt}=k_{3}\cdot \;ES\;(t), \end{array}$$

where *k*_1_, *k*_2_, *k*_3_, are the system unknown parameters. Let *x*_1_(*t*), *x*_2_(*t*), *x*_3_(*t*)and *x*_4_(*t*) represent *S*(*t*),*E*(*t*), *ES*(*t*) and *P*(*t*) respectively. Then, the above equation can be rewritten into

(16) $$\begin{array}{l} {\dot{x}}_{1}=-k_{1}x_{1}x_{2}+k_{2}x_{3},\\ {\dot{x}}_{2}=-k_{1}x_{1}x_{2}+k_{2}x_{3}+k_{3}x_{3},\\ {\dot{x}}_{3}=k_{1}x_{1}x_{2}-k_{2}x_{3}-k_{3}x_{3},\\ {\dot{x}}_{4}=k_{3}x_{3},\\ \Rightarrow \;\left[\begin{array}{c} {\dot{x}}_{1}\\ {\dot{x}}_{2}\\ {\dot{x}}_{3}\\ {\dot{x}}_{4} \end{array}\right]\;=\;\left[\begin{array}{ccc} -x_{1}x_{2} & x_{3} & 0\\ -x_{1}x_{2} & x_{3} & x_{3}\\ x_{1}x_{2} & -x_{3} & -x_{3}\\ 0 & 0 & x_{3} \end{array}\right]\;\cdot \left[\begin{array}{c} k_{1}\\ k_{2}\\ k_{3} \end{array}\right] \end{array}$$

Then, the mass balance constraint becomes:

(17) $$\left\{ \begin{array}{l} x_{2}(t)+x_{3}(t)=x_{2}(t_{0})+x_{3}(t_{0})\\ x_{1}(t)+x_{3}(t)+x_{4}(t)=x_{1}(t_{0})+x_{3}(t_{0})+x_{4}(t_{0}) \end{array}\right.$$

Or in matrix form, we have

(18) $$A_{eq}\text{·}x={\text{b}}_{eq}$$

where $A_{eq}=\left[\begin{array}{cccc} 0 & 1 & 1 & 0\\ 1 & 0 & 1 & 1 \end{array}\right]\;$ and $b_{eq}=\left[\begin{array}{c} x_{2}(t_{0})+x_{3}(t_{0})\\ x_{1}(t_{0})+x_{3}(t_{0})+x_{4}(t_{0}) \end{array}\right]$.

According to (16), we have

(19) $$\begin{array}{l} \Phi (x(t))=\;\left[\begin{array}{ccc} -x_{1}x_{2} & x_{3} & 0\\ -x_{1}x_{2} & x_{3} & x_{3}\\ x_{1}x_{2} & -x_{3} & -x_{3}\\ 0 & 0 & x_{3} \end{array}\right],\;\theta =\;\left[\begin{array}{c} k_{1}\\ k_{2}\\ k_{3} \end{array}\right],\\ \text{and }\hat{\Psi }(\hat{x}(t_{i}))=\int_{t=t_{0}}^{t_{i}}\left[\begin{array}{ccc} -{\hat{x}}_{1}{\hat{x}}_{2} & {\hat{x}}_{3} & 0\\ -{\hat{x}}_{1}{\hat{x}}_{2} & {\hat{x}}_{3} & {\hat{x}}_{3}\\ {\hat{x}}_{1}{\hat{x}}_{2} & -{\hat{x}}_{3} & -{\hat{x}}_{3}\\ 0 & 0 & {\hat{x}}_{3} \end{array}\right]\;dt. \end{array}$$

An artificial data set with four time courses was created. A total of 40 sampling points were assigned on each time courses. The observation data was perturbed by a zero mean Gaussian white noise *η*(*t*) ~ N(0, σ^2^) in order to simulate the observation error. The estimated state $\hat{x}$ was computed by solving the quadratic programming sub-problem A_1_. The unknown parameter values were estimated using P_2_. The searching region of the parameter values was [0, +∞). All the computations were performed on a Pentium Dual Core computer (2.13 GHz ×2) with 2 GB RAM. The algorithm was implemented with Matlab-7 using the interior point algorithm. To quantify the fitness of the estimated model, the following relative squared error (RSE) measure *J *is employed:

(20) $$J=\frac{1}{N\cdot n}\sum_{i=1}^{n}\sum_{j=0}^{N}{\left(\frac{{\hat{x}}_{i}(t_{j})-x_{i}(t_{j})}{x_{i}(t_{j})}\right)}^{2},$$

where ${\hat{x}}_{i}(t_{j})$ is the estimated time-course at time *t_j _*of a state variable *x_i_*, and *x_i_*(*t_j_*) represents the "true" time-courses without noise at time *t_j_*. Note that smaller RSE *J *reflects better estimation. In order to obtain a statistical result on the quality of the estimation, 5,000 trials were performed. At each trial an estimated $\hat{\theta }$ is computed using P_2_. Then, the mean estimation and standard deviation were deduced. The computation was very efficient and only took a few seconds for one estimation trial.

**Figure 1:**
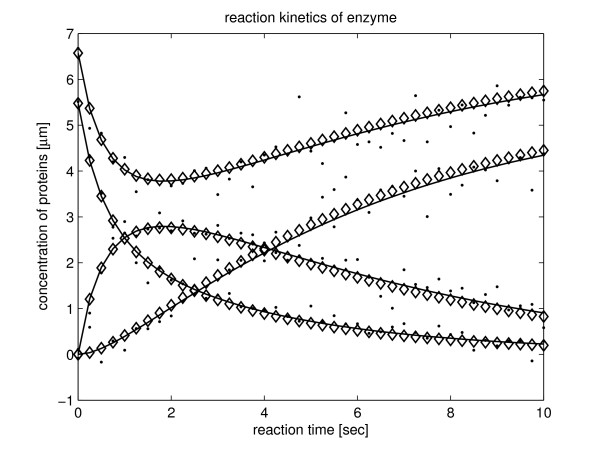
**The dynamic profiles of a trial with observation data subject to 10% random noises**. Solid lines represent the "true" time-series data without noise, dots represent the measured time-series data with added artificial noise, and diamonds represent the estimated time-series data produced by the model.

Table 1 shows the statistical results of the estimation. It reveals that all the system parameter values are estimated successfully with a relative error of around 0.01% in noise free condition. When the system is subjected to a 10% observation noise level, all the mean estimated parameter values are within a relative tolerance, better than 2%. The RSE between the time-courses, produced by inferred model, and the given time-series data, averaged smaller than 1% even subject to 10% observation noise level condition. For this reason, we have confidence that the proposed method is robust within ±10% noise ratio (more complicated models and trials on P_2 _can be found in part I of additional file 1). Figure 1 shows the responses of one trial.

**Table 1:** Statistical results of parameter estimation of enzyme kinetic model.

	Nominal Value		Mean estimation ± standard deviation	
		
		Noise level: 0%	Noise level: 5%	Noise level: 10%
*k*_1_	0.18	0.1796 ± 33.5081e-6	0.1794 ± 0.0008	0.1808 ± 0.0025
*k*_2_	0.20	0.1993 ± 108.4891e-6	0.1968 ± 0.0031	0.1963 ± 0.0106
*k*_3_	0.23	0.2300 ± 1.5946e-6	0.2326 ± 0.0001	0.2345 ± 0.0004
*J*		6.8620e-8 ± 7.7337e-8	1.0996e-4 ± 9.3270e-5	3.0328e-4 ± 3.2160e-4

### The mammalian G1/S transition network model

Next, the mammalian G1/S transition network model, which includes a set of proteins and regulatory gene network, is used to test P_3_. In the mammalian G1/S transition network, pRB and AP-1 are the tumor suppressor from the family of pocket proteins and the family of transcription factors that mediate mitogenic signals, E2F1 is the transcription factor targeting genes that regulate cell cycle progression, Cyclin D/cdk4,6, cyclin E/cdk2, complexes characterizing the G1- and S- phases. There are various positive and negative feedback loops in the network controlling the G1/S transition. The positive feedback regulation of E2F1 and a double activator-inhibitor module can lead to bistability. The double activator-inhibitor module of the antagonistic plays E2F/DP on pRB make up the key unit of this phase transition. The graph representation of the mammalian G1/S transition network model can be found in additional file 1 and more details can refer to Swat et al. [5]. Definition of Variables for G1/S Transition Model is shown in Table 2. The corresponding ODE model is as follows

**Table 2:** Definition of Variables for G1/S Transition Model.

Symbol	*x*_1_	*x*_2_	*x*_3_	*x*_4_	*x*_5_	*x*_6_	*x*_7_	*x*_8_	*x*_9_
Acronym	*pRB*	*E2F1*	*CycD_i_*	*CycD_a_*	*AP *- 1	*pRB_p_*	*pRB_p_p*	*CycE_i_*	*CycE_a_*

(21) $$\begin{array}{l} {\dot{x}}_{1}=k_{1}\frac{x_{2}}{K_{m1}+x_{2}}\frac{J_{11}}{J_{11}+x_{1}}\frac{J_{61}}{J_{61}+x_{6}}-k_{16}x_{1}x_{4}+k_{61}x_{6}-\phi_{1}x_{1},\\ {\dot{x}}_{2}=k_{p}+k_{2}\frac{a_{2}^{2}x_{2}^{2}}{K_{m2}^{2}+x_{2}^{2}}\frac{J_{12}}{J_{12}+x_{1}}\frac{J_{62}}{J_{62}+x_{6}}-\phi_{2}x_{2},\\ {\dot{x}}_{3}=k_{3}x_{5}+k_{23}x_{2}\frac{J_{13}}{J_{13}+x_{1}}\frac{J_{63}}{J_{63}+x_{6}}+k_{43}x_{4}-k_{34}x_{3}\frac{x_{4}}{K_{m4}+x_{4}}\\ \;\;\;\;\;\;\;\;\;\;-\phi_{3}x_{3},\\ {\dot{x}}_{4}=k_{34}x_{3}\frac{x_{4}}{K_{m4}+x_{4}}-k_{43}x_{4}-\phi_{4}x_{4},\\ {\dot{x}}_{5}=F_{m}+k_{25}x_{2}\frac{J_{15}}{J_{15}+x_{1}}\frac{J_{65}}{J_{65}+x_{6}}-\phi_{5}x_{5},\\ {\dot{x}}_{6}=k_{16}x_{1}x_{4}-k_{61}x_{6}-k_{67}x_{6}x_{9}+k_{76}x_{7}-\phi_{6}x_{6},\\ {\dot{x}}_{7}=k_{67}x_{6}x_{9}-k_{76}x_{7}-\phi_{7}x_{7},\\ {\dot{x}}_{8}=k_{28}x_{2}\frac{J_{18}}{J_{18}+x_{1}}\frac{J_{68}}{J_{68}+x_{6}}+k_{98}x_{9}-k_{89}x_{8}\frac{x_{9}}{K_{m9}+x_{9}}-\phi_{8}x_{8},\\ {\dot{x}}_{9}=k_{89}x_{8}\frac{x_{9}}{K_{m9}+x_{9}}-k_{98}x_{9}-\phi_{9}x_{9}, \end{array}$$

where *x *is the set of state variables. There are totally 9 states and 39 parameters. The nominal parameter values are shown in Table 3.

**Table 3:** Results of parameter estimation of the mammalian G1/S transition network model.

Parameters	Nominal value	Estimated parameters			
		Noise level 0%	Noise level: 2.5%	Noise level: 5%	Noise level: 10%
*k*_1_	1	0.9957	1.0150	1.1105	1.6037
*k*_2_	1.6	1.5989	1.4138	1.5187	1.0315
*k*_3_	0.05	0.0500	0.0528	0.0392	0.0381
*k*_16_	0.4	0.4002	0.4440	0.3959	0.9331
*k*_34_	0.04	0.0400	0.0414	0.0337	0.0215
*k*_43_	0.01	0.0100	0.0142	0.0090	1.45e-10
*k*_61_	0.3	0.2985	0.3432	0.2847	0.8185
*k*_67_	0.7	0.6999	0.4535	1.3974	1.3108
*k*_76_	0.1	0.0999	0.0457	0.2446	0.1845
*k*_23_	0.3	0.1219	0.4134	0.6132	0.5579
*k*_25_	0.9	0.1785	0.7063	0.8291	0.7874
*k*_28_	0.06	0.0601	0.0669	0.0222	0.0198
*k*_39_	0.07	0.0700	0.0549	0.0520	0.0334
*k*_96_	0.01	0.0100	0.0441	0.0002	4.55e-14
*a*	0.04	0.0400	0.1257	0.1260	0.1265
*J*_11_	0.5	0.4992	0.5612	0.4252	0.6523
*J*_12_	5	5.0025	4.8940	4.6892	5.4021
*J*_15_	0.001	0.0051	0.0011	0.0010	0.0011
*J*_18_	0.6	0.5990	0.7253	0.8014	1.1290
*J*_61_	5	5.2581	4.1474	6.4585	7.2003
*J*_62_	8	8.0088	29.734	39.403	41.408
*J*_65_	6	5.9222	8.7804	9.3474	7.8076
*J*_68_	7	6.9916	31.979	25.125	36.795
*J*_13_	0.002	0.0050	0.0013	0.0016	2.61e-14
*J*_63_	2	1.9740	1.4726	0.4203	19.871
*K*_m1_	0.5	0.4905	0.5267	0.5601	0.0410
*K*_m2_	4	3.9985	4.0482	4.1061	3.8495
*K*_m4_	0.3	0.2999	0.2838	0.2735	0.2338
*K*_m9_	0.005	0.0054	3.69e-5	2.03e-5	3.88e-6
*K_p_*	0.05	0.0499	0.0452	0.0496	0.0311
*ϕ*_1_	0.005	0.0044	0.0057	0.0041	0.0073
*ϕ*_2_	0.1	0.0999	0.0920	0.0983	0.0693
*ϕ*_3_	0.023	0.0230	0.0261	0.0164	0.0152
*ϕ*_4_	0.03	0.0300	0.0279	0.0253	0.0218
*ϕ*_5_	0.01	0.0100	0.0098	0.0101	0.0101
*ϕ*_6_	0.06	0.0606	0.0627	0.0608	0.1518
*ϕ*_7_	0.04	0.0401	0.0436	0.0404	0.0788
*ϕ*_8_	0.06	0.0600	0.1546	0.0024	0.0260
*ϕ*_9_	0.05	0.0500	0.0025	0.0439	0.0276
*J*		7.5399e-6e	0.0005	0.0009	0.0025

**Figure 2:**
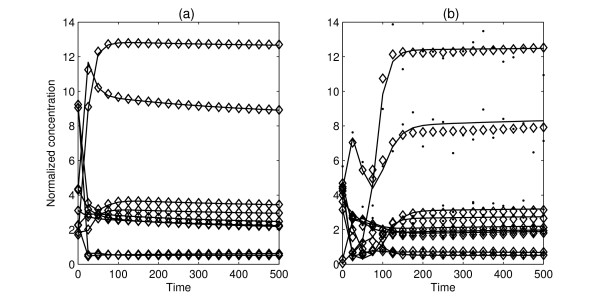
**The dynamic profiles of two trials**. Solid lines represent the "true" time-series data without noise, dots represent the measured time-series data with added artificial noise, and diamonds represent the estimated time-series data produced by the model: (a) noise free condition (b) 10% random noise condition.

Here, P_3 _was solved by the Stochastic Raking Evolution Strategy (SRES) algorithm [35]. The searching region of the parameters was [0, 50*θ *]. SRES uses stochastic ranking as the constraint handling technique, which adjusts the balance between the objective and penalty functions automatically during the evolutionary search. The observation data include 4 sets of time course, which consists of 40 sample points. For trials with noise free data, the algorithm converged in 8 ~ 9 hours after 250,000 ~ 300,000 iterations. The estimated parameter values, as shown in Table 3 are almost identical to the nominal parameter values. However, for *k*_23_, *k*_25 _and *J*_15_, the estimated values are far from the nominal values, but the RSE measure is almost zero, which possibly implies that the system is insensitive with the changes of *k*_23_, *k*_25 _and *J*_15_. This phenomenon reveals that the G1/S transition model either has some parameters that are insensitive to the chosen observation, or they are non-identifiable parameters [36,37]. It is worth mentioning that the this large computational effort is the consequence of the very tight convergence criteria, an almost equal good result can be reached within 200,000 generations in about 6.5 hours with the RSE measure *J *is smaller than 1%. Figure 2(a) shows the "true" time-series data without noise and computed dynamic time-series data from one identified model. When 10% random noises are added, the convergence time increased and the relative estimation errors between estimated parameters and nominal parameters increased with the increase of noise. However, the time-series produced by the estimated model is very similar to the original data, namely the RSE *J *is still small. This phenomenon may imply that there is no need to estimated every parameters accurately to achieve a model with equivalent dynamical properties with a good degree of accuracy. As the simulation time is long, performing thousands of simulations as the first method in order to evaluate the mean and variance of estimated parameters is impractical. Thus, due to the lack of space, results of just a few selected trial are shown in Table 3 (more trial results can be found in additional file 1).

Trials were performed using Matlab-7. The main reason to use Matlab is that it is a convenient environment to visualize all the information arising from the optimization runs of the solver, evaluate new algorithms and modify existing algorithms. In contrast to the convenience, it is worth mentioning that Matlab programs usually are one order of magnitude (10 times or more) slower than equivalent compiled Fortan or C codes [19]. This is the major drawbacks of carrying programs out with Matlab. However, even in this situation, the performance of the proposed methods is acceptable.

For fair comparison, we also used the SRES algorithm to solve the same parameter estimation problem in the same searching region, but using NLP-P_0 _with differential algebraic constraints as cost function. In this condition, after running 1 day, the algorithm failed to produce a set of parameters that can produce reasonable simulation result. We further reduced the searching region to [0, 3*θ *] and used noise free data, but the estimation result was still not good and the RSE *J *is larger than 10.

**Figure 3:**
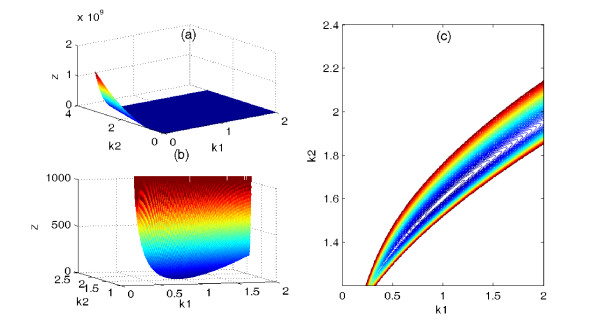
**Cost function surface and contours**. (Color online) (a) Cost function surface of the P_0 _as parameters *k*_1 _and *k*_2 _are varied; (b) displays the same data as (a) on the expanded scale; (c) corresponding contours near the nominal parameter value.

Here, we use the G1/S model to show the differences of the cost function surfaces between NLP-P_0 _and NLP-P_3_: in this case, the cost function of P_0 _is a highly irregular and complicated manifold with multiple local minima; the augmented cost function adopted in problem P_3 _is a much "smoother" function and hence it is easier for the NLP algorithm to converge to the solution. In order to simplify the analysis for exposition purpose, we only vary parameters *k*_1 _and *k*_2 _over the range *k*_1 _ϵ [0; 2 ] and *k*_2 _ϵ [0, 3.2 ] and fix all other parameters at their nominal values. Figure 3(a) displays the cost function surface of P_0 _, while Figure 3(b) exhibits the same data as Figure 3(a) on the expanded scale and Figure 3(c) is the corresponding contour plots. Figure 3(a) shows that the cost function surface of is a ridge, which drops suddenly from 10^9 ^to 0. However, Figure 3(b) reveals the cost function surface of P_0 _are actually banana-shaped valley around the nominal value of the fixed parameters, this unfavorable profile can slow down the convergence rate of the algorithm. Furthermore, there are many local minima in the banana-shaped valley. Some algorithms, such as simulated annealing, genetic algorithm, have been proposed to overcome these problem. However, these algorithms are all computationally demanding. In conclusion, these cost function features make the problem P_0 _a severe challenge to every optimization algorithm.

**Figure 4:**
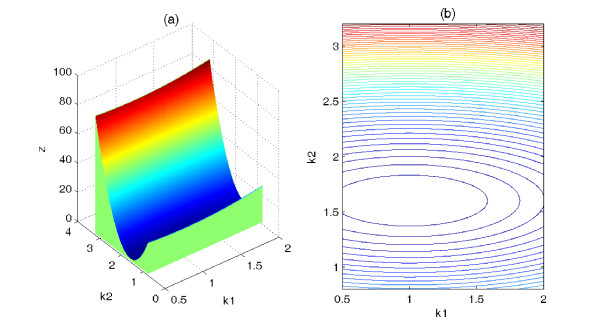
**Cost function surface and contours**. (Color online) Cost function surface of the P_3 _as parameters *k*_1 _and *k*_2 _are varied; (b) corresponding contours of the cost function.

With the same condition, Figure 4(a) displays the cost function surface of P_3_, while Figure 4(b) shows the corresponding contour line. Compared with the cost function surface of P_0_, the cost function surface of P_3 _is bowl-shaped, which is smoother. Similar results has also been observed when other combination of parameters served as variables. Obviously, if all 39 parameters vary at the same time, the surface of the cost function will be more "uneven" and more complicated. However, in this case, from the previous observations, the cost function surface of P_3 _is smoother than the cost function surface of P_0._

Furthermore, P_3 _only involves algebraic equations as objective function and constraints. These properties make the NLP-P_3 _easier to solve.

## Discussion and Conclusion

In this paper, two parameter estimation methods based on spline theory are proposed. One aims at a narrower class of systems which is linear in parameters; however, it can cover many commonly found biological systems. The benefit is that the estimation problem can be transformed in an LP sub-algorithm which are fast and robust. Additional linear constraints can be embedded relative easily. For general systems, the problem is solved by an NLP with algebraic constraints, which is more computationally demanding.

A simple enzyme kinetic model and the mammalian G1/S transition network model were used as benchmarks to evaluate the performance of the two proposed methods. We illustrate the usefulness with more examples in additional files 1 but these do not remotely cover all the conditions.

During the simulation of the mammalian G1/S transition network model, we found that the estimated parameter set Φ*_A _*≡ {*k*_1_, *k*_2_, *k*_*p*_, *J*_11_, *J*_12_, *K*_*m*1_, *K*_*m*2_, *ϕ*_1_, *ϕ*_2_} were well within the respective nominal values. While the set Φ_*B *_≡ {*J*_61_, *J*_62_, *J*_63_, *J*_65_, *J*_68_} were far from their nominal values. However, the time-series produced by the estimated model were very similar to the original data. This phenomena reveals that some parameter values are insensitive in the searching region. Interestingly, we find that the "sensitive" or "easily identified" parameters set Φ_*A *_are also the parameters of the double-activator-inhibitor module of the antagonistic players E2F/DP and pRB, which makes up the core unit of the G1/S transition model [5]. This phenomenon may imply that the parameter values of the core module are sensitive and easy to identify. In contrast, the parameters set Φ_*B *_seems to be insensitive, which may reflect that *pRG_p_*(*x*_6_) is not a key element of the total system. However, to identify which parameter values or variables are important, a sensitivity analysis is needed [38], which is another important topic in systems biology and deserves a more detailed study. This sensitivity analysis is a pre-process for isolating those states and parameters which are sensitive in order to reduce the dimension of the system model and to improve the numerical stability for the core estimation problem.

For most biological systems, the ODE models are often high-dimensional and nonlinear. The problem of system parameter estimation is computationally expensive and can easily be trapped in local minima. We find that under noisy conditions, it is almost impossible to accurately estimate every parameter of the sloppy biological system model. However, in practice, a model with equivalent dynamical properties with a good degree of accuracy can be constructed based on dominant sensitive parameters and system states. The following are some of practical observations:

1. High quality experiment data is essential for identifying accurate biology systems. When the experiment data is corrupted with high level noise, it needs more experimental data. If an insufficient amount of time-series data is given as observed profiles, the high degree-freedom of systems biology models ensures that many candidate solutions will be found.

2. Perform a sensitivity analysis and identifiability analysis before the identification phase [36-38].

3. For systems models with insensitive or non-identifiable parameters, the search may lead to a solution where some parameters can have large deviation, but still produce satisfactory system responses. This problem can be partly relieved by introducing auxiliary information (additional constraints such as shrinking the searching region) of the model into the algorithm. However, it remains difficult to be solved completely by improving parameter estimation strategy. It indicates that researchers should focus on predictions rather than on accurately estimating every parameter.

Although the proposed algorithms are fast and robust, there is certainly room for improvement: for method 1, it is not general enough to catch every case; for method 2, the price for the simplicity and generality is at the expansion of the optimization variable dimension. Under high noise condition, method 2 is still not robust enough. At the moment, the testing is based on Matlab which is much slower than native codes produced by C, Fortran, etc, however the conversion is straight forward. Currently, many high-speed computation engines are available that make use of parallelism, for instance multi-cluster engines, array-processing engines etc. Hence, one possible way is developed algorithm on these high-speed computation engines environment. Another possible way is developing hybrid algorithms to incorporate elements from evolution algorithms such as GA, SA and PSO. In this paper, we have considered the parameter estimation problem with known structure. However, it is easy to expand our method to structure identification by introducing an additional penalty term to the objective function [39].

## Authors' contributions

All authors contributed to the development of the theory and analysis of the results. CJ designed, analyzed and implemented the algorithm, performed the experiments and wrote the main body of manuscript. LY contributed to the development of the method and writing the manuscript and providing the critical review of the manuscript. All authors have read and approved the final version of the manuscript.

## Supplementary Material

Additional file 1 **In this additional file, we tested the proposed methods on seven systems biology models were used to test: TNFα -Mediated NF-*κ*B-Signaling Pathway Model, RKIP Regulated ERK Pathway model and the model of irreversible inhibition of HIV proteinase; Yeast fermentation pathway Model, large-scale target genetic network model, a three step pathway model and the mammalian G1/S transition network model**. Click here for file

Additional file 2 **In this additional file, we use E2F/DP dimmer model to illustrate the differences between the three different type methods mentioned in the paper: (i) the direct optimization method; (ii) decomposition methods; (iii) methods Combine spline theory and NLP**. Click here for file
