# Supplementary material for: Parameter estimation in systems biology models using spline approximation
Source: BMC Syst Biol. 2011 Jan 24;5:14. doi: 10.1186/1752-0509-5-14 (PMC3750107; doi:10.1186/1752-0509-5-14)
Supplement: Additional file 1 — In this additional file, we tested the proposed methods on seven systems biology models were used to test: TNFα -Mediated NF-κB-Signaling Pathway Model, RKIP Regulated ERK Pathway model and the model of irreversible inhibition of HIV proteinase; Yeast fermentation pathway Model, large-scale target genetic network model, a three step pathway model and the mammalian G1/S transition network model. [file 1752-0509-5-14-S1.PDF]

Supplementary Material

Biological systems always have many reactants, cells, enzymes and reaction pathways. Hence, the ODE model always has many variant states and equations. The problem of estimating parameters of biological systems now attracts a lot of attention [3-6]. However, it is still a bottleneck of computational analysis of biological systems and is the most challenging task. Thus, it is reasonable to use biological systems testing the proposed method. We tested the proposed methods on seven systems biology models in this supplementary material and the experimental results are given. The experiments were executed in a Pentium Dual Core computer (2.13GHz×2) with 2 GB RAM and the computation just use one core. All the algorithms are coded in Matlab-7.

I. Trials on LP-spline method

Three models, TNFα -Mediated NF-κB-Signaling Pathway Model, RKIP Regulated ERK Pathway model and the model of irreversible inhibition of HIV proteinase, are used to test the LP method P_3. We find the LP method is efficient, which just needs a few seconds to finish the identification procedures. The trial results also show that the LP-spline method is robust to noise.

1. TNFα -Mediated NF-κB-Signaling Pathway

The graph representation of TNFα-Mediated NF-κB-Signalling Pathway Model is shown in Fig. S1. More details can be found in [1].

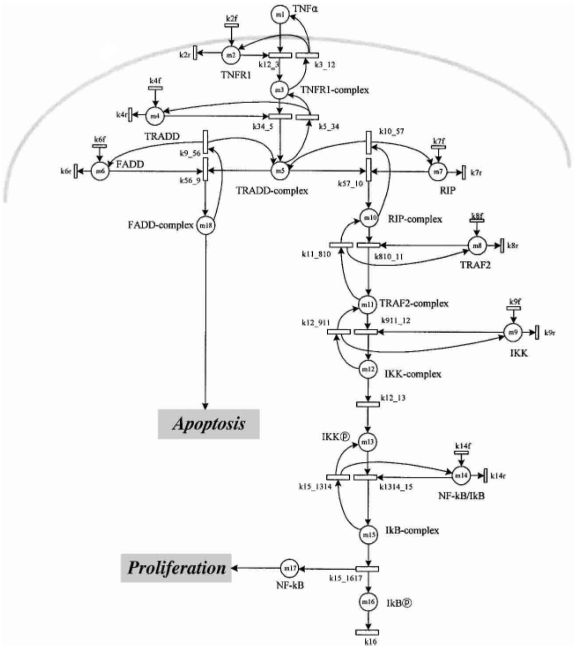

Fig. S1. Graphical model of the TNF α -Mediated NF- κ B-Signaling Pathway

The corresponding ODE model is shown as follows

$$
\begin{aligned}
\dot{x}_1 &= -k_{12\_3}\cdot x_1\cdot x_2 + k_{3\_12}\cdot x_3\\
\dot{x}_2 &= k_{2f} - k_{2r}\cdot x_2 - k_{12\_3}\cdot x_1\cdot x_2 + k_{3\_12}\cdot x_3\\
\dot{x}_3 &= k_{12\_3}\cdot x_1\cdot x_2 - (k_{3\_12} + k_{34\_5}\cdot x_4)\cdot x_3 + k_{5\_34}\cdot x_5\\
\dot{x}_4 &= k_{5\_34}\cdot x_5 - (k_{4r} + k_{34\_5}\cdot x_3)\cdot x_4 + k_{4f}\\
\dot{x}_5 &= k_{34\_5}\cdot x_3\cdot x_4 - (k_{56\_9}\cdot x_6 + k_{57\_10}\cdot x_7 + k_{6f} + k_{5\_34})\cdot x_5 + k_{9\_56}\cdot x_{18} + k_{10\_57}\cdot x_{10}\\
\dot{x}_6 &= -(k_{6r} + k_{56\_9}\cdot x_5)\cdot x_5 + k_{9\_56}\cdot x_{18}\\
\dot{x}_7 &= k_{7f} - (k_{7r} + k_{57\_10}\cdot x_5)\cdot x_7 + k_{10\_57}\cdot x_{10}\\
\dot{x}_8 &= -(k_{8r} + k_{810\_11}\cdot x_{10})\cdot x_8 + k_{11\_810}\cdot x_{11} + k_{8f}\\
\dot{x}_9 &= -(k_{9r} + k_{911\_12}\cdot x_{11})\cdot x_9 + k_{12\_911}\cdot x_{12} + k_{9f}\\
\dot{x}_{10} &= k_{57\_10}\cdot x_5\cdot x_7 - k_{810\_11}\cdot x_8\cdot x_{10} + k_{11\_810}\cdot x_{11}\\
\dot{x}_{11} &= k_{810\_11}\cdot x_8\cdot x_{10} + k_{12\_911}\cdot x_{12} - k_{911\_12}\cdot x_9\\
\dot{x}_{12} &= k_{911\_12}\cdot x_{11} - (k_{12\_13} + k_{12\_911})\cdot x_{12} + k_{11\_910}\cdot x_{11}\\
\dot{x}_{13} &= k_{15\_1314}\cdot x_{15} + k_{12\_13}\cdot x_{12} - k_{1314\_15}\cdot x_{13}\cdot x_{14}\\
\dot{x}_{14} &= k_{14f} + k_{15\_1314.}\cdot x_{15} - (k_{14f} + k_{1314\_15}\cdot x_{13})\cdot x_{14}\\
\dot{x}_{15} &= k_{1314\_15}\cdot x_{13}\cdot x_{14} - (k_{15\_1314} + k_{15\_1617})\cdot x_{15}\\
\dot{x}_{16} &= k_{15\_1617}\cdot x_{15} - k_{16}\cdot x_{16}\\
\dot{x}_{17} &= k_{15\_1617}\cdot x_{15}\\
\dot{x}_{18} &= k_{56\_9}\cdot x_6 - k_{9\_56}\cdot x_{18}
\end{aligned}
\tag{S1.}
$$

where x is the set of variant state and k is the set of parameters. There are totally 18 states and 31 parameters.

The same as trials on enzyme kinetic model, the algorithm is carried out with Matlab 7 using the interior point algorithm. We use the relative squared error (RSE) measure J_1

$$J_1 = \frac{1}{N\cdot n}\sum_{i=1}^{n}\sum_{j=0}^{N}\left(\frac{\hat{x}_i(t_j) - x_i(t_j)}{x_i(t_j)}\right)^2 \tag{S2}$$

and the relative power error (RPE) measure J_2

$$J_2 = \frac{\sum_{i=1}^{n}\sum_{j=0}^{N}(\hat{x}_i(t_j) - x_i(t_j))^2}{\sum_{i=1}^{n}\sum_{j=0}^{N}x_i(t_j)^2}, \tag{S3.}$$

to quantify the fitness of the estimated model. $\hat{x}_i(t_j)$ is the estimated time-course at time t_j of a state variable x_i, and $x_i(t)$ represents the "true" time-courses at time t_j of x_i. Note that smaller RSE J_1 and RPE J_2 reflect better estimation. In order to

obtain a statistical result, the trials are performed 5,000 times. Then, the mean estimation and standard deviation were deduced. The computation was found to be very efficient and only took a few seconds for one estimation trial.

Table S1. Statistical Results of Parameter Estimation of TNFα -Mediated NF-κB-Signalling Pathway

	True Value	**Mean estimation ± standard deviation**		
		**Noise level : 0%**	**Noise level : 5%**	**Noise level : 10%**
k12_3	0.001	0.001 ± 92.7070e-15	0.0010 ± 17.9177e-9	0.0010 ± 78.9046e-9
k3_12	0.004	0.004 ± 10.8683e-12	0.0041 ± 1.5254e-6	0.0043 ± 6.5875e-6
k34_5	0.001	0.001 ± 43.5975e-15	0.0010 ± 4.7391e-9	0.0011 ± 16.1612e-9
k5_34	0.004	0.004 ± 50.8086e-12	0.0050 ± 25.6835e-6	0.0071 ± 80.2357e-6
k56_9	0.001	0.001 ± 161.9326e-15	0.0097 ± 71.6390e-9	0.0010 ± 288.3438e-9
k9_56	0.004	0.004 ± 985.7952e-12	0.0039 ± 197.8220e-9	0.0040 ± 777.3115e-9
k57_10	0.001	0.001 ± 200.2562e-15	0.0097 ± 70.1668e-9	0.0010 ± 267.6990e-9
k10_57	0.004	0.004 ± 9.5858e-12	0.0036 ± 734.5330e-9	0.0032 ± 2.7788e-6
k810_11	0.001	0.001 ± 11.3751e-15	0.00099 ± 4.2362e-9	0.0010 ± 14.2936e-9
k11_810	0.004	0.004 ± 191.3661e-15	0.0042 ± 57.2591e-9	0.0039 ± 190.0612e-9
k911_12	0.001	0.001 ± 22.5953e-15	0.0010 ± 9.0648e-9	0.0010 ± 31.4437e-9
k12_911	0.004	0.004 ± 21.1893e-12	0.0043 ± 10.3363e-6	0.0058 ± 32.0643e-6
k12_13	0.1	0.1 ± 4.6036e-12	0.0993 ± 9.6190e-6	0.0989 ± 46.0233e-6
k1314_15	0.001	0.001 ± 5.1685e-15	0.00097 ± 4.3691e-9	0.0009 ± 18.5849e-9
k15_1314	0.004	0.004 ± 561.8335e-15	0.0038 ± 609.3980e-9	0.0032 ± 2.4151e-6
k15_1617	0.004	0.004 ± 30.4936e-15	0.0039 ± 31.2931e-9	0.0040 ± 124.3286e-9
k16	0.1	0.1 ± 2.2802e-12	0.1001 ± 12.2088e-6	0.1004 ± 47.2196e-6
k2f	0.14	0.1399 ± 171.4398e-9	0.1379 ± 2.4239e-3	0.1560 ± 8.6559e-3
k2r	0.006	0.006 ± 903.5406e-12	0.0058 ± 13.8580e-6	0.0070 ± 49.1608e-6
k4f	0.14	0.14 ± 2.8670e-9	0.1440 ± 691.4118e-6	0.1546 ± 2.2217e-3
k4r	0.006	0.006 ± 31.5977e-12	0.0064 ± 8.4016e-6	0.0071 ± 26.9883e-6
k6f	0.14	0.1399 ± 244.5216e-12	0.1377 ± 83.5578e-6	0.1362 ± 311.1926e-6

k6r	0.006	0.006 ± 650.0673e-15	0.0059 ± 197.4582e-9	0.0060 ± 688.1909e-9
k7f	0.14	0.1399 ± 272.1748e-9	0.1337 ± 3.0086e-3	0.1386 ± 9.8736e-3
k7r	0.006	0.006 ± 528.5854e-12	0.0055 ± 6.6590e-6	0.0054 ± 20.4037e-6
k8f	0.14	0.14 ± 40.64417e-9	0.1417 ± 3.8078e-3	0.1571 ± 13.0690e-3
k8r	0.006	0.006 ± 2.0280e-9	0.0062 ± 18.5583e-6	0.0074 ± 62.0856e-6
k9f	0.14	0.14 ± 650.5820e12	0.1390 ± 109.0057e-6	0.1442 ± 509.0036e-6
k9r	0.006	0.006 ± 6.5069e-12	0.0062 ± 3.9424e-6	0.0069 ± 17.4581e-6
k14f	0.14	0.14 ± 658.5134e-12	0.1430 ± 490.3568e-6	0.1564 ± 1.9932e-3
k14r	0.006	0.006 ± 7.9040e-12	0.0066 ± 8.3988e-6	0.0084 ± 35.0765e-6
J1		0 ± 7.9732e-18	0.0006 ± 110.0882e-9	0.0022 ± 1.7733e-6
J2		0 ± 114.7066e-18	0.0008 ± 138.3083e-9	0.003 ± 2.0360e-6

Table S1. shows the Statistical Results of the estimation. It reveals that all the system parameters were estimated successfully with a relative error around 0.01% in noise free conditions and the RSE J_1 and RPE J_2 are almost 0. When the system is subjected to a 10% observation noise level, all the mean estimated parameters were within a relative tolerance better than 1%. The RSE between the time-courses, produced by inferred model and the given time-series data, averaged smaller than 1% even subject to 10% observation noise level condition. Fig. S2.-S4. show one trail result with different noise level, 0%, 5% and 10%, respectively.

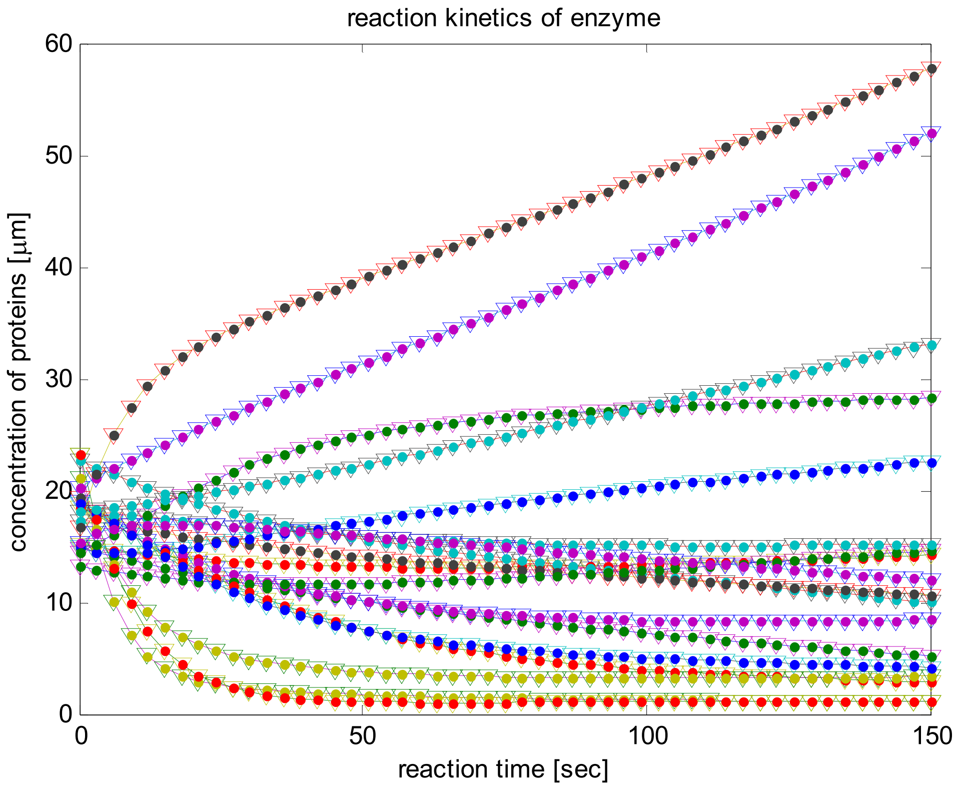

Fig. S2. One experiment result with 0%: solid lines represent original data, dots represent the measured data with noise, and triangles represent estimate data.

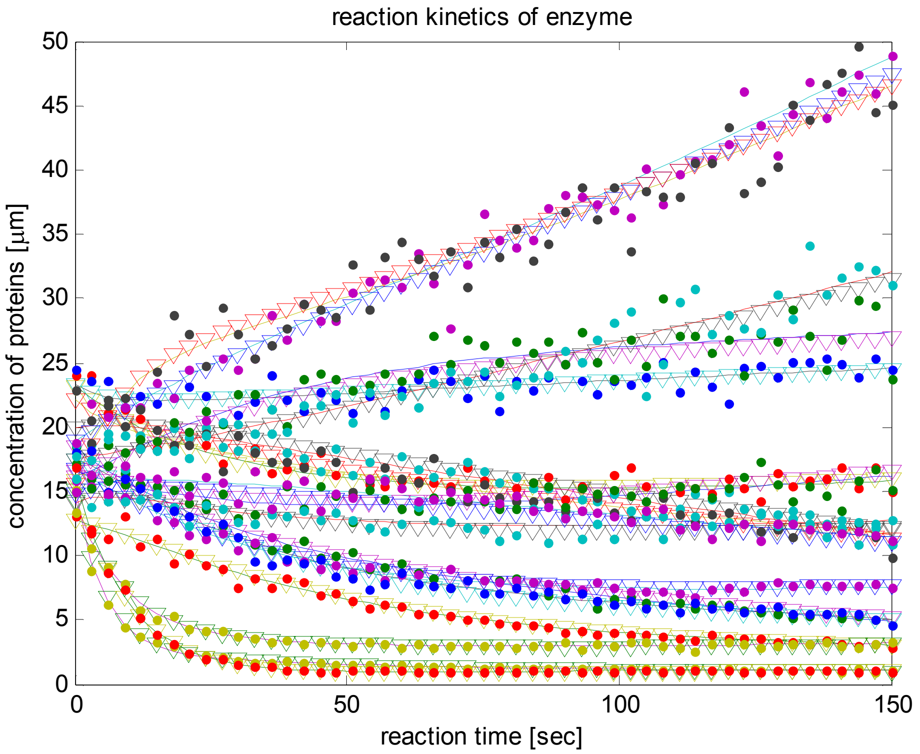

Fig. S3. One experiment result with 5%: solid lines represent original data, dots represent the measured data with noise, and triangles represent estimate data.

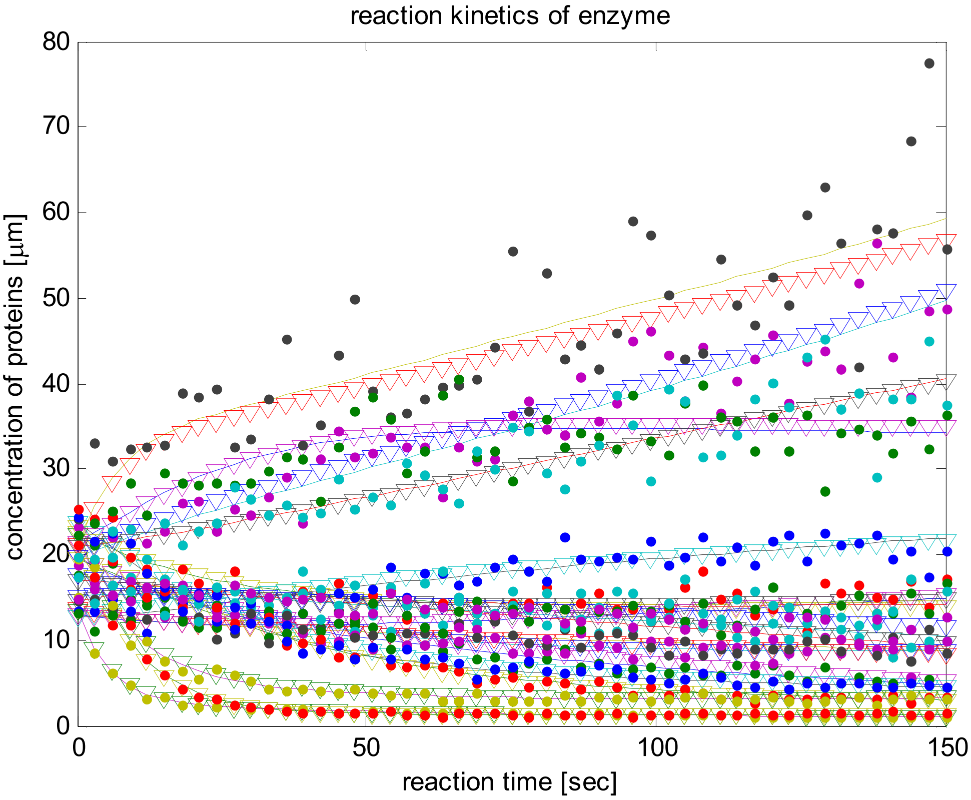

Fig. S4. One experiment result with 10%: solid lines represent original data, dots represent the measured data with noise, and triangles represent estimate data.

2. RKIP Regulated ERK Pathway model

The RKIP regulated ERK signaling pathway [2], as shown in Figure S5, is a circle representing a state for the concentration of a protein, e.g. a circle with x1 denotes the concentration of the activated protein Raf-1; a rectangular bar contains kinetic parameters which denote the reaction rates with respect to corresponding reactions; an edge with arrow connecting a circle and a bar indicates the direction of a signal flow.

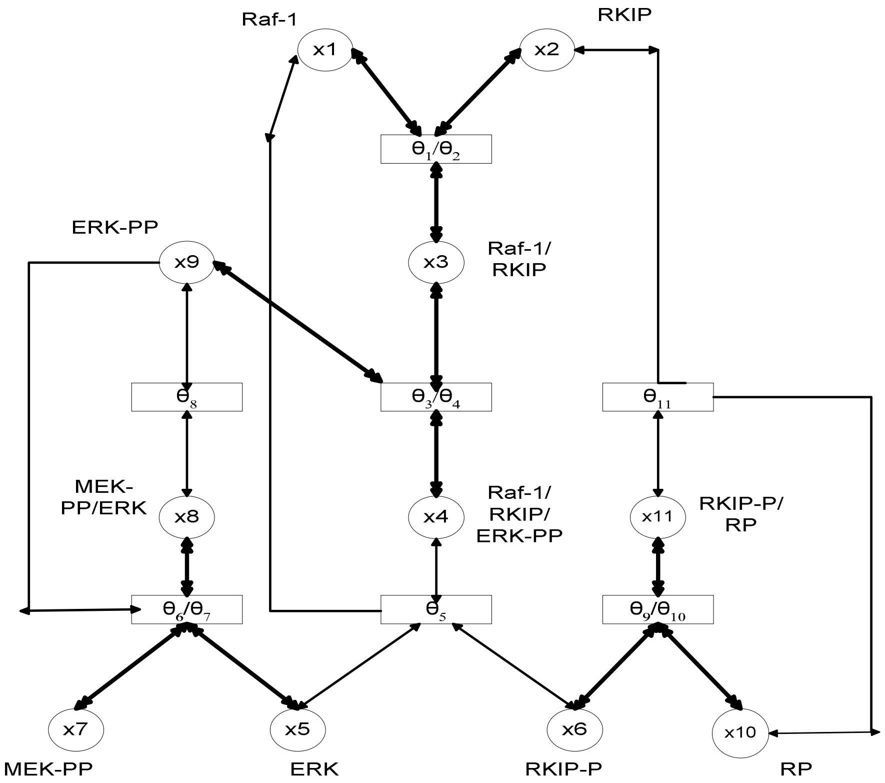

Fig. S5. Graphical representation of ERK signaling pathway regulated by RKIP

The corresponding ODE model is shown as following

$$
\begin{aligned}
\dot{x}_1 &= -k_1 x_1 x_2 + k_2 x_3 + k_5 x_4 \\
\dot{x}_2 &= -k_1 x_1 x_2 + k_2 x_3 + k_{11} x_{11} \\
\dot{x}_3 &= k_1 x_1 x_2 - k_2 x_3 - k_3 x_3 x_9 + k_4 x_4 \\
\dot{x}_4 &= k_3 x_3 x_9 - k_4 x_4 - k_5 x_4 \\
\dot{x}_5 &= k_5 x_4 - k_6 x_5 x_7 + k_7 x_8 \\
\dot{x}_6 &= k_5 x_4 - k_9 x_6 x_{10} + k_{10} x_{11} \\
\dot{x}_7 &= -k_6 x_5 x_7 + k_7 x_8 + k_8 x_8 \\
\dot{x}_8 &= k_6 x_5 x_7 - k_7 x_8 - k_8 x_8 \\
\dot{x}_9 &= -k_3 x_3 x_9 + k_4 x_4 + k_8 x_8 \\
\dot{x}_{10} &= -k_9 x_6 x_{10} + k_{10} x_{11} + k_{11} x_{11} \\
\dot{x}_{11} &= k_9 x_6 x_{10} - k_{10} x_{11} - k_{11} x_{11}
\end{aligned}
\tag{S4.}
$$

There are totally 11 states and 11 parameters. In order to obtain a statistical result, the trials are performed 5,000 times. Then, the mean estimation and standard deviation are deduced. The computation was found to be very efficient and only took a few seconds for one estimation trial.

Table S2. Statistical Results of Parameter Estimation of RKIP Regulated ERK Pathway

model

	True Value	Mean estimation ± standard deviation		
		Noise level : 0%	Noise level : 5%	Noise level : 10%
k1	0.53	0.5300 ± 8.2638e-11	0.5271 ± 0.0003	0.5190 ± 0.0017
k2	0.0072	0.0068 ± 3.8702e-7	0.0169 ± 0.0008	0.0134 ± 0.0010
k3	0.625	0.6249 ± 3.4765e-11	0.6188 ± 0.0006	0.5971 ± 0.0030
k4	0.00245	0.0021 ± 1.6454e-8	0.0083 ± 0.0001	0.0067 ± 0.0001
k5	0.0315	0.0315 ± 5.0994e-9	0.0498 ± 0.0070	0.0491 ± 0.0099
k6	0.8	0.8000 ± 2.0761e-10	0.7721 ± 0.0020	0.7042 ± 0.0088
k7	0.0075	0.0075 ± 1.4866e-6	0.0077 ± 0.0005	0.0169 ± 0.0009
k8	0.071	0.0709 ± 9.3742e-7	0.1317 ± 0.0461	0.108 ± 0.0503
k9	0.92	0.9199 ± 1.1935e-9	0.9237 ± 0.0051	0.9239 ± 0.0203
k10	0.00122	0.0013 ± 1.7747e-7	0.0059 ± 0.0000	0.0066 ± 0.0000
k11	0.87	0.8693 ± 1.1826e-6	0.8257 ± 0.1391	0.7745 ± 0.4637
J1		0 ± 8.8934e-21	0 ± 1.4325e-9	0.0001 ± 28.0334e-9
J2		0 ± 4.2121e-15	0.0304 ± 1.8648e-3	0.0991 ± 0.0034

Table S1. shows the Statistical Results of the estimation. It reveals that all the system parameters were estimated successfully with a relative error around 0.1% in noise free conditions and the RSE J_1 and RPE J_2 are almost 0. When the system is subjected to a 10% observation noise level, all the mean estimated parameters were within a relative tolerance better than 3%. Fig. S6. shows one trail result with 10% noise level.

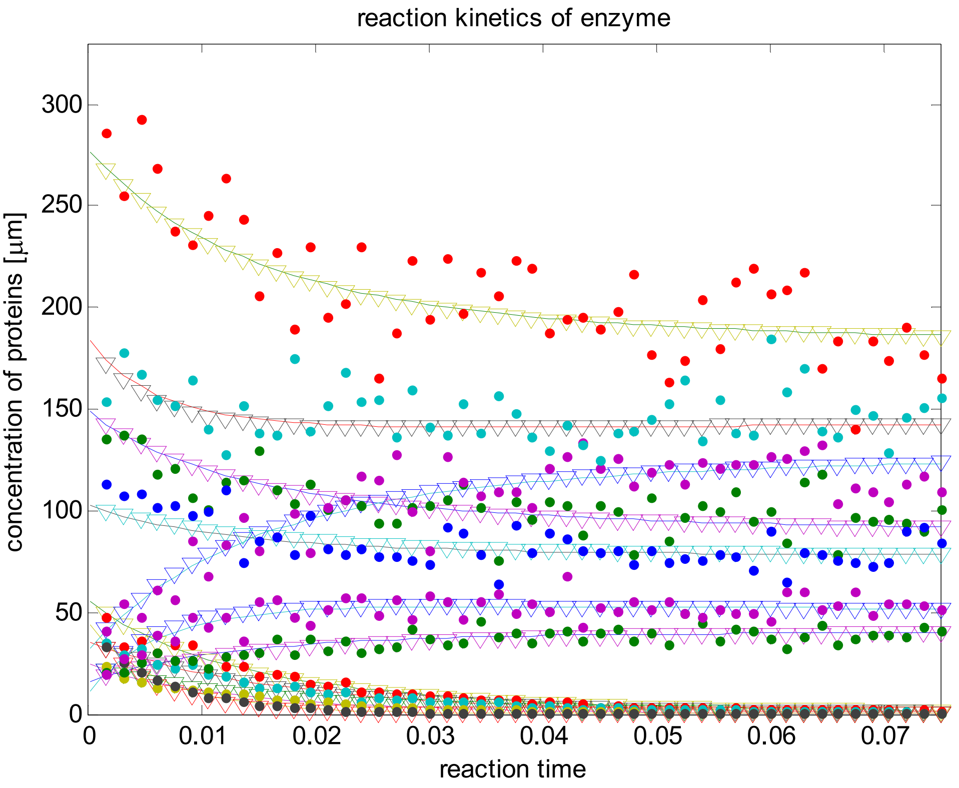

Fig. S6. One experiment result with 10%: solid lines represent original data, dots represent the measured data with noise, and triangles represent estimate data.

3. Mechanism of irreversible inhibition of HIV Proteinase [7][8]

In this example, we estimate the rate constants of the mechanism of irreversible inhibition of HIV proteinase [7]. Graph representation is shown is Fig. 7. The corresponding ODE model is equation S5. Data from 10 sets of time courses were used for this analysis. Gaussian white noise was added to the observation data. Statistic results are shown in Table S3.

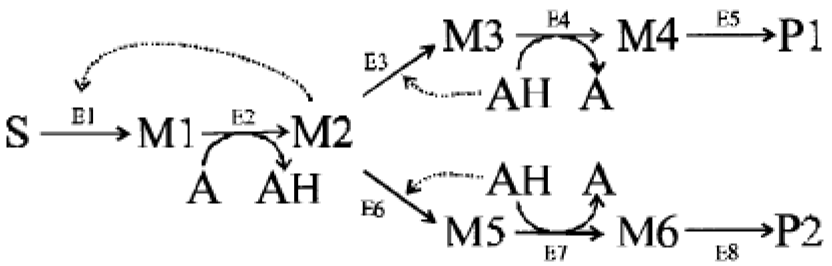

Fig. S7. Graphical representation of the irreversible inhibition of HIV Proteinase model

$$
\begin{aligned}
\dot{x}_1 &= -2k_{md}x_1^2 + 2k_{dm}x_7 \\
\dot{x}_2 &= k_r x_5 - k_{on}x_2x_7 + k_p x_6 \\
\dot{x}_3 &= -k_{on}x_3x_7 + k_s x_5 \\
\dot{x}_4 &= -k_{on}x_4x_7 + k_i x_8 \\
\dot{x}_5 &= k_{on}x_3x_7 - k_s x_5 - k_r x_5 \\
\dot{x}_6 &= k_{on}x_2x_7 - k_p x_6 \\
\dot{x}_7 &= k_a x_1^2 - k_d x_7 - k_{on}x_3x_7 + k_s x_5 + k_r x_5 - k_{on}x_2x_7 + k_p x_6 - k_{on}x_4x_7 + k_i x_8 \\
\dot{x}_8 &= k_{on}x_4x_7 - k_i x_8 - k_{de}x_8 \\
\dot{x}_9 &= k_{de}x_8
\end{aligned}
\tag{S5.}
$$

There are totally 9 states and 10 parameters. $x_2, x_3, x_4, x_5, x_6, x_8, x_9$ satisfied mass balance constraint, which means

$$C = x_2(t) + x_3(t) + x_4(t) + x_5(t) + x_6(t) + x_8(t) + x_9(t)$$

where $C = x_2(t_0) + x_3(t_0) + x_4(t_0) + x_5(t_0) + x_6(t_0) + x_8(t_0) + x_9(t_0)$ is constant. In order to obtain a statistical result, the trials are performed 5,000 times. Then, the mean estimation and standard deviation were deduced. The computation was found to be very efficient and only took a few seconds for one estimation trial. The trial results are very similar to the trials on TNFα-Mediated NF-κB-Signaling Pathway Model and RKIP Regulated ERK Pathway model. Hence, we do not give any further discussion. Fig. S7. show one trail result with 10% noise level.

Table S3. Statistical Results of Parameter Estimation of the irreversible inhibition of HIV proteinase model

	True Value	Mean estimation ± standard deviation		
		Noise level : 0%	Noise level : 5%	Noise level : 10%
ka	0.1	0.1 ± 703.3498e-12	0.0798 ± 1.9656-3	0.055 ± 3.5489e-3
kd	0.01	0.01 ± 12.6717e-12	0.0103 ± 32.7390-6	0.0131 ± 75.7749-6
ki	0.05	0.05 ± 15.2907e-12	0.0336 ± 21.2072-6	0.0058 ± 36.8131e-6
kde	0.1	0.1 ± 193.8411e-15	0.1001 ± 2.2678-6	0.0995 ± 8.0543e-6
kon	0.8	0.8 ± 742.0685e-12	0.6948 ± 782.7544-6	0.5076 ± 2.6007e-3

ks	0.001	0.00106 ± 2.6227e-9	0 ± 0	0 ± 0
kp	2.5	2.5 ± 2.5309e-9	2.3673 ± 6.4335-3	2.1776 ± 16.2318e-3
kmd	3	3.0001 ± 10.6335e-9	2.6088 ± 11.1409-3	1.9158 ± 38.1556e-3
kdm	0.1	0.1 ± 1.1587e-15	0.0993 ± 26.3104-6	0.0985 ± 78.3392e-6
k10	0.005	0.005 ± 19.5528e-18	0.0049 ± 423.8135-9	0.0047 ± 1.4699e-6
J1		0 ± 12.7228e-21	0.0004 ± 214.7404-9	0.0034 ± 7.0853e-6
J2		0 ± 136.8436e-21	0.0016 ± 1.7674-6	0.0185 ± 230.8570e-6

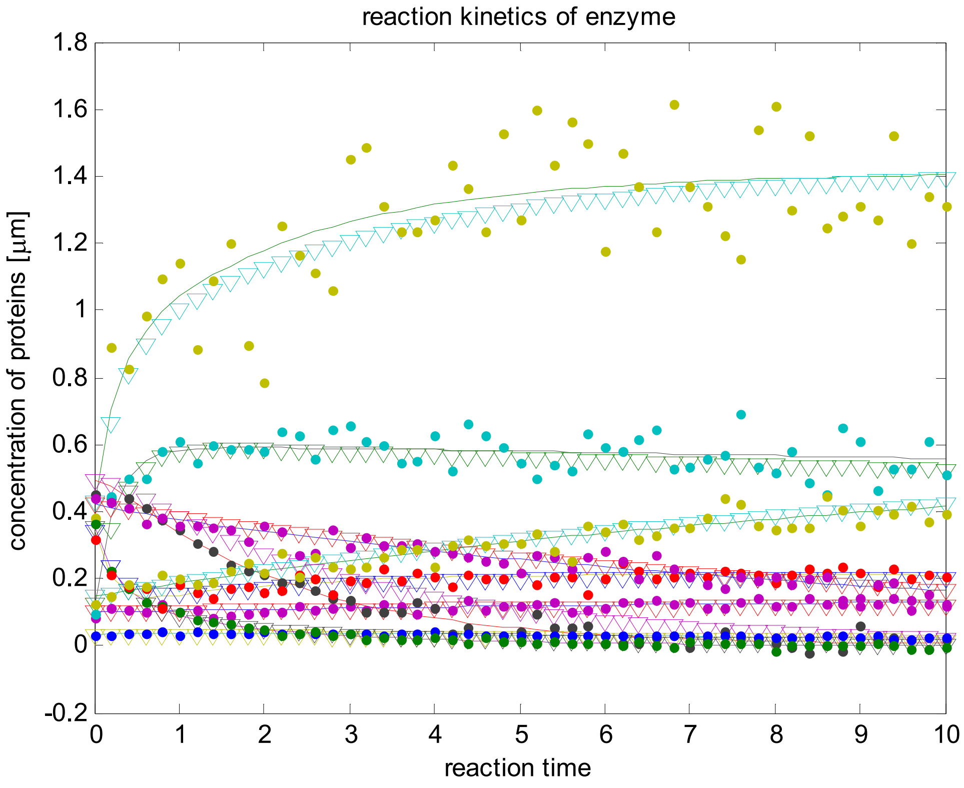

Fig. S7. One experiment result with 10%: solid lines represent original data, dots represent the measured data with noise, and triangles represent estimate data.

II. Trials on NLP-Spline method

The next four models, Yeast fermentation pathway Model [9,10], a target genetic network model [13,14], a three step pathway model [12] and the mammalian G1/S transition network model [11], are used to test the NLP-spline method P_3.

4. Yeast fermentation pathway

Here, we consider an S-system model of the ethanol production by yeast, which is an example of real biochemical networks and has been extensively studied [9,10]. This model refers to anaerobic and non-growing conditions with glucose as the sole carbon source in the absence of nitrogen. As shown in Fig. S8., this metabolic pathway involves five dependent variables and eight independent variables with steady-state value.

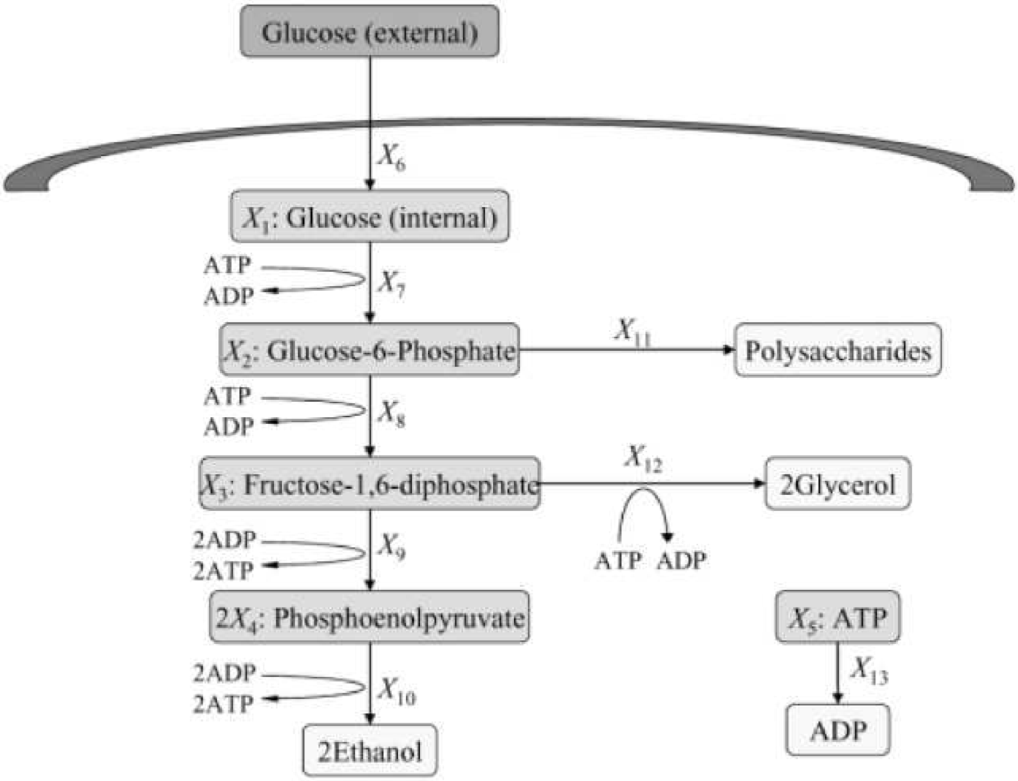

Fig. S8. Illustration of the anaerobic fermentation pathway in an yeast.

The corresponding ODEs model is equation S6.

$$\dot{x}_1 = 1.0006 x_2^{-0.0492} x_6 - 1.6497 x_1^{0.5582} x_5^{0.0465} x_7$$
$$\dot{x}_2 = 1.6497 x_1^{0.5582} x_5^{0.0465} x_7 - 0.5793 x_2^{0.5097} x_5^{-0.2218} x_8^{0.8322} x_{11}^{0.1678}$$
$$\dot{x}_3 = 0.4536 x_2^{0.4407} x_5^{-0.2665} x_8 - 0.2456 x_3^{0.4506} x_4^{0.0441} x_5^{0.092} x_9^{0.8547} x_{12}^{0.1453}$$
$$\dot{x}_4 = 0.2365 x_3^{0.5285} x_5^{0.0994} x_9 - 2.0892 x_3^{-0.0075} x_4^{0.304} x_5^{0.0484} x_{10}$$
$$\dot{x}_5 = 1.406 x_3^{0.2605} x_4^{0.152} x_5^{0.0739} x_9^{0.5} x_{10}^{0.5} - 2.9437 x_1^{0.1962} x_2^{0.1791} x_5^{0.2354} x_7^{0.3514} x_8^{0.2925} x_{11}^{0.0589} x_{13}^{0.297} \quad \text{(S6.)}$$

This metabolic pathway involves five dependent variables, glucose, glucose-6-phosphate, frutose1, 60diphosphate, phosphoenolpyruvate and ATP, and

eight independent variables with steady-state value, glucose uptake, hexokinase, phosphofructorkinase, glyceraldehyde 3-phosphate dehydrogenase, pyruvate kinase, polysaccharide storage, glycerol production and ATPase. Definition of Variables for Yeast fermentation pathway model is shown in Table S4.

Table S4. Definition of Variables for Yeast fermentation pathway model

<table>
<tr><td colspan="20">Dependent variables</td></tr>
<tr><td colspan="4">Glucose</td><td colspan="4">Gluocose-6-phophate</td><td colspan="4">Fructose-1,6-diphosphate</td><td colspan="4">phosphoenolpyruvate</td><td colspan="4">ATP</td></tr>
<tr><td colspan="4">x_1</td><td colspan="4">x_2</td><td colspan="4">x_3</td><td colspan="4">x_4</td><td colspan="4">x_5</td></tr>
<tr><td colspan="20">Independent variables</td></tr>
<tr><td colspan="5">Glucose uptake</td><td colspan="5">hexokinase</td><td colspan="5">phosphofructokinase</td><td colspan="5">glyceraldehyde</td></tr>
<tr><td colspan="5">x_6</td><td colspan="5">x_7</td><td colspan="5">x_8</td><td colspan="5">x_9</td></tr>
<tr><td colspan="5">47.5 mM min^{-1}</td><td colspan="5">24.1 mM min^{-1}</td><td colspan="5">53.9 mM min^{-1}</td><td colspan="5">91.4 mM min^{-1}</td></tr>
<tr><td colspan="5">Pyruvate kinase</td><td colspan="5">Polysaccharide storage</td><td colspan="5">Glycerol production</td><td colspan="5">ATPase</td></tr>
<tr><td colspan="5">x_{10}</td><td colspan="5">x_{11}</td><td colspan="5">x_{12}</td><td colspan="5">x_{13}</td></tr>
<tr><td colspan="5">18.1 mM min^{-1}</td><td colspan="5">82.9 mM min^{-1}</td><td colspan="5">95.4 mM min^{-1}</td><td colspan="5">1.0 mM min^{-1}</td></tr>
</table>

There are totally 33 unknown parameter values need to be estimated. In [9], a genetic programming method is used to infer the S-system model of this biochemical network. The computation was performed in the Linux system with AMD Athlon MP 2800+ processor and 2GB memory and one experiment needs about 79h. Here, we choice Simulation Annealing (SA) algorithm and the identification program is implemented on Matlab 7 in a 2.13GHz CPU. The searching regions were [0.0 3.0] for α_i and β_i, [-1.0 1.0] for $g_{i,j}$ and $h_{i,j}$. For trial with noise free data, the algorithm converges in about 5.5 hours after 1,000,000 iterations. If one increase the iteration times, the result will be better. One can balance between the time consuming and accuracy. As the noise level increase, the convergence time increases and the convergence rate decreases. When a noise of 10% of the standard deviation is added, the convergence time is around 7 hours. As the computation environment is different, directly comparison is not fair. However, the experiment results show our method actually can give a satisfied result in a reasonable computation time.

As the simulation time is long, we can not do thousands times of simulations as the trials on the LP method and calculate the mean and variance. As a few dozens trials may not reflect the stochastic property, we just gives a few results in different noise level in **Table S5-S7**. The results show that even in 10% noise condition, the relative squared error (RSE) measure J_1 and the relative power error (RPE) measure J_2 is small, which $J_1 \approx 0.02$ and $J_2 \approx 0.05$. One should notice that some estimated parameters are always far from their nominal value, such as g_{25} and h_{43}. And in high noise condition, the deviation of estimated parameters is large, but the identified system still produces satisfactory system responses. This phenomenon implies that the Yeast fermentation pathway model may be sloppy system.

Table S5. Nominal parameter values and some experimental results of Parameter Estimation of the Yeast fermentation pathway model in noise free condition

Parameters	Nominal value	Estimated parameters (noise level : 0%)					
α_1	1.0006	1.0135	0.9939	1.0638	1.0032	1.0725	1.058
g_{12}	-0.0492	-0.0425	-0.0496	-0.0422	-0.0496	-0.0432	-0.047
β_1	1.6497	1.6874	1.6365	1.7691	1.6533	1.7978	1.7588
h_{11}	0.5582	0.5497	0.5625	0.5302	0.5578	0.5327	0.533
h_{15}	0.0465	0.0458	0.0469	0.0455	0.046	0.0428	0.0441
α_2	1.6497	1.3465	1.8968	1.7054	1.6273	1.2895	2.1216
g_{21}	0.5582	0.5758	0.5472	0.56	0.5598	0.5976	0.4835
g_{25}	0.0465	0.1281	0.0088	0.0328	0.0518	0.1135	-0.0014
β_2	0.5793	0.4857	0.689	0.5913	0.5698	0.4583	0.7714
h_{22}	0.5097	0.5194	0.4554	0.5211	0.5114	0.551	0.4384
h_{25}	-0.2218	-0.1574	-0.2393	-0.2354	-0.2165	-0.1859	-0.2378
α_3	0.4536	0.2672	0.4288	0.3766	0.4482	0.4975	0.7014
g_{32}	0.4407	0.5603	0.464	0.5042	0.4406	0.4394	0.2864
g_{35}	-0.2665	-0.2348	-0.2875	-0.3378	-0.2609	-0.2919	-0.1462
β_3	0.2456	0.1519	0.2315	0.1927	0.2449	0.2774	0.3934

h_{33}	0.4506	0.5431	0.4685	0.4995	0.4496	0.4359	0.3063
h_{34}	0.0441	0.0566	0.0475	0.0541	0.0447	0.0498	0.0363
h_{35}	0.092	0.1919	0.0903	0.1015	0.0935	0.0554	0.0832
α_4	0.2365	0.2029	0.154	0.2088	0.2587	0.198	0.2569
g_{43}	0.5285	0.5631	0.5877	0.5431	0.5193	0.533	0.5166
g_{45}	0.0994	0.0612	0.0818	0.0725	0.0576	0.1248	0.035
β_4	2.0892	2.0095	1.6033	1.9677	2.28	1.8581	2.338
h_{43}	-0.0075	-0.0784	-0.1302	-0.0642	-0.0207	-0.0535	-0.0485
h_{44}	0.304	0.3604	0.3964	0.3362	0.3069	0.3294	0.3213
h_{45}	0.0484	-0.0053	0.0358	0.0283	0.0092	0.0648	-0.0185
α_5	1.406	1.2455	1.3113	1.1684	1.4011	1.3524	0.7005
g_{53}	0.2605	0.2713	0.271	0.2792	0.2611	0.2658	0.3624
g_{54}	0.152	0.1632	0.1628	0.1761	0.1517	0.1548	0.2829
g_{55}	0.0739	0.0977	0.0752	0.0907	0.0752	0.0803	0.1421
β_5	2.9437	2.54	2.6474	2.3526	2.9439	2.8365	0.9362
h_{51}	0.1962	0.2111	0.2335	0.2174	0.196	0.1992	0.3431
h_{52}	0.1791	0.183	0.1864	0.1969	0.1798	0.1825	0.3595
h_{53}	0.2354	0.277	0.2537	0.2675	0.2347	0.2402	0.4351
J_1		0.0007	0.0005	0.0003	0.0000	0.0004	0.0045
J_2		0.0008	0.0009	0.0008	0.0000	0.0005	0.0069

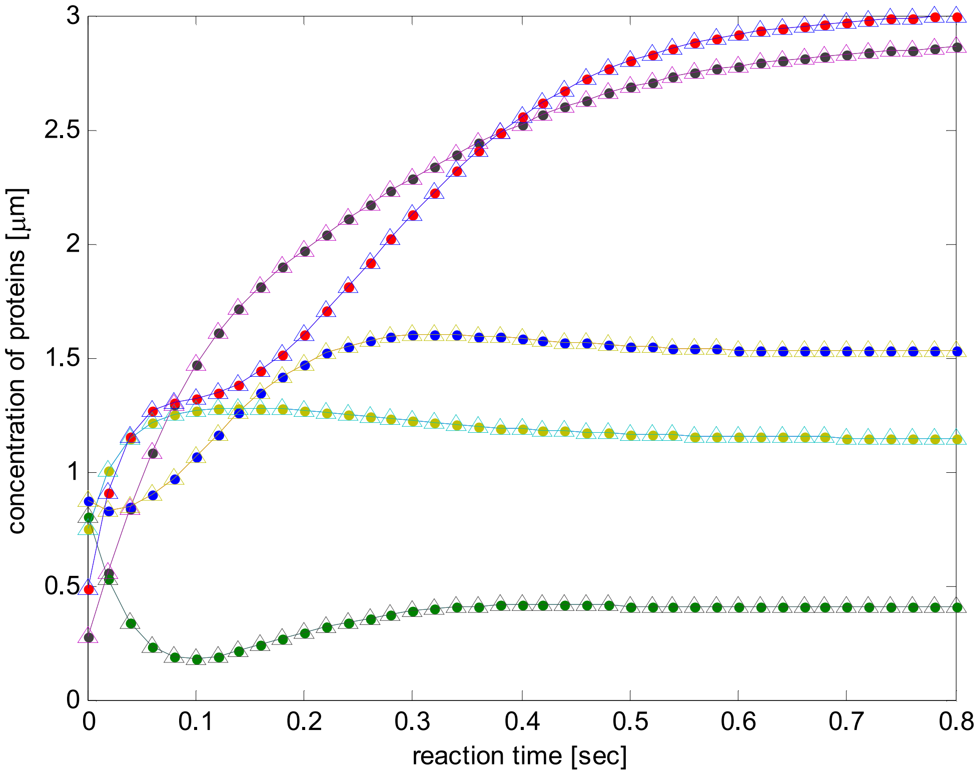

Fig. S9. The dynamic profiles of a trial with observation data subject to a noise level of 0% of standard deviation: solid lines represent "true" time-series data, dots represent the measured time-series data with added artificial noise, and triangles represent estimate time-series data produced by inferred model.

Table S6. Nominal parameter values and some experimental results of Parameter Estimation of Yeast fermentation pathway model with observation data with 5% noise

Parameters	Nominal Value	Estimated parameters (noise level: 5%)					
α_1	1.0006	1.079	0.9047	0.6142	0.6999	0.5386	1.1932
g_{12}	-0.0492	-0.065	-0.0896	-0.3271	-0.1715	-0.2852	-0.0543
β_1	1.6497	1.7812	1.5144	0.9564	1.1487	0.7026	2.0872
h_{11}	0.5582	0.53	0.4309	0.3764	0.5669	0.9973	0.3573
h_{15}	0.0465	0.0354	0.0207	-0.1312	-0.0368	-0.0118	0.0061
α_2	1.6497	1.1975	2.1385	1.4793	1.5704	1.3652	1.2655
g_{21}	0.5582	0.8209	0.4811	0.2127	0.0924	0.5748	0.3782
g_{25}	0.0465	-0.1206	-0.0435	0.1776	0.1468	-0.0571	0.2812
β_2	0.5793	0.3726	0.8088	0.4818	0.506	0.4721	0.4008

h_{22}	0.5097	0.7677	0.3604	0.3337	0.3827	0.4513	0.4123
h_{25}	-0.2218	-0.4995	-0.2528	0.1015	0.0005	-0.2554	0.1913
α_3	0.4536	1.858	0.8736	1.7196	0.4358	0.1809	1.177
g_{32}	0.4407	0.1327	0.2669	0.0258	0.5109	0.5615	0.0917
g_{35}	-0.2665	-0.0358	-0.1488	-0.0452	-0.072	-0.3062	0.0369
β_3	0.2456	1.0678	0.4938	1.0206	0.1998	0.0818	0.7064
h_{33}	0.4506	0.1391	0.2719	0.0436	0.5908	0.6345	0.1339
h_{34}	0.0441	0.0083	0.0273	0.039	-0.0545	0.0746	0.0596
h_{35}	0.092	0.0691	0.0575	-0.0079	0.3412	0.2721	0.1024
α_4	0.2365	0.1945	0.2391	0.2896	0.246	0.1332	0.1194
g_{43}	0.5285	0.6011	0.3656	-0.3557	0.2683	0.7598	0.0584
g_{45}	0.0994	-0.1335	0.0541	0.4874	0.1206	-0.0992	0.2279
β_4	2.0892	2.0822	2.1119	1.9038	1.824	1.6142	1.079
h_{43}	-0.0075	-0.2031	-0.1272	-0.7095	-0.2011	-0.2484	-0.7045
h_{44}	0.304	0.434	0.3108	0.1455	0.2362	0.5313	0.3132
h_{45}	0.0484	-0.138	0.0202	0.5247	0.1452	-0.0776	0.2941
α_5	1.406	0.7282	0.9954	1.1704	0.898	0.6948	1.6736
g_{53}	0.2605	0.3877	0.5149	0.0553	0.2943	0.5207	0.1331
g_{54}	0.152	0.1788	0.2732	0.2657	0.3283	0.423	0.5315
g_{55}	0.0739	0.1416	-0.2204	0.0127	0.0319	-0.2539	-0.327
β_5	2.9437	1.3978	1.5229	2.0641	1.241	0.6723	1.8364
h_{51}	0.1962	0.1608	0.0883	0.2619	0.2533	0.6773	0.7244
h_{52}	0.1791	0.2696	0.3761	-0.2174	0.2909	0.1519	-0.3269
h_{53}	0.2354	0.3227	0.0793	0.4647	0.3413	0.4549	0.4083
J_1		0.0009	0.0017	0.0015	0.0018	0.0018	0.0010
J_2		0.0035	0.0025	0.0062	0.0042	0.0056	0.0039

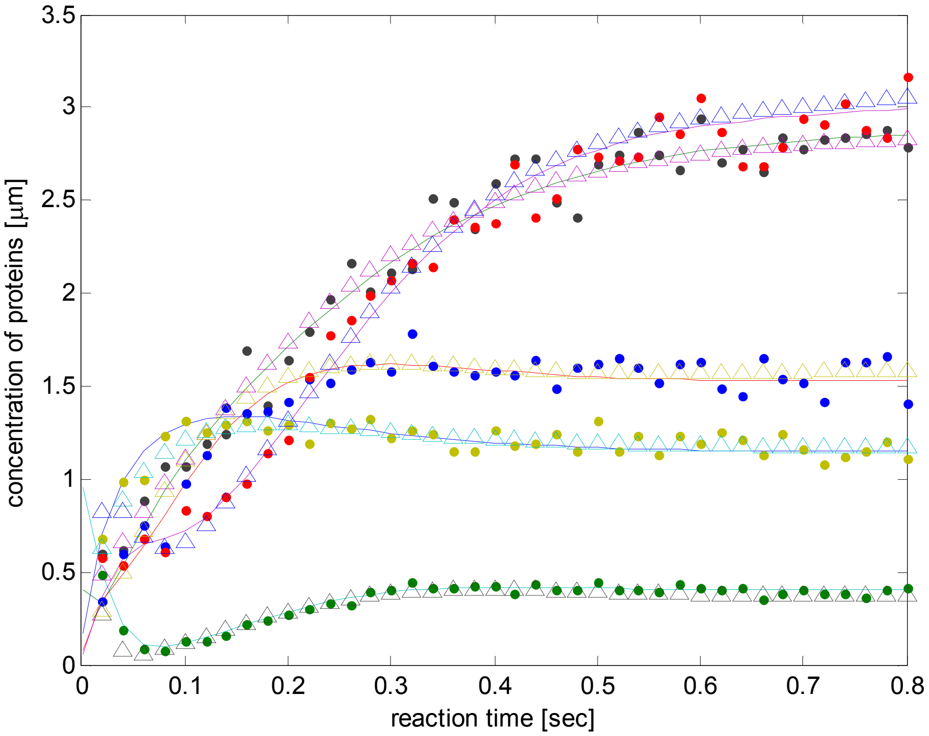

Fig. S10. The dynamic profiles of a trial with observation data subject to a noise level of 5% of standard deviation: solid lines represent "true" time-series data, dots represent the measured time-series data with added artificial noise, and triangles represent estimate time-series data produced by inferred model.

Table S7. Nominal parameter values and some experimental results of Parameter Estimation of Yeast fermentation pathway model with observation data subject to a noise level of 10% noise level condition

Parameters	True value	Estimated parameters Noise level : 10%					
α_1	1.0006	0.9804	0.6601	0.5295	1.2814	0.8185	1.3583
g_{12}	-0.0492	-0.212	-0.2151	-0.4177	-0.0977	-0.2696	-0.2681
β_1	1.6497	1.4705	0.8959	0.6579	2.186	1.2607	2.1144
h_{11}	0.5582	0.694	0.7857	0.9388	0.3958	0.7859	0.4939
h_{15}	0.0465	-0.038	0.0329	-0.0615	0.0057	-0.1055	-0.1066
α_2	1.6497	1.167	1.8278	1.81	1.8758	1.4336	1.843
g_{21}	0.5582	0.8711	0.7336	0.5699	0.503	0.7203	0.4958

g_{25}	0.0465	-0.2134	-0.4216	-0.2585	-0.1439	-0.054	0.0113
β_2	0.5793	0.4322	0.686	0.6028	0.6793	0.4711	0.6573
h_{22}	0.5097	0.296	0.6202	0.4937	0.4776	0.5813	0.3994
h_{25}	-0.2218	-0.2917	-0.7981	-0.4679	-0.4423	-0.3156	-0.1719
α_3	0.4536	0.6497	1.2343	0.1993	0.3617	0.8066	1.3904
g_{32}	0.4407	0.1764	0.1912	0.5975	0.3699	0.2581	0.1944
g_{35}	-0.2665	-0.2351	-0.2975	-0.0324	-0.1145	-0.1965	-0.4979
β_3	0.2456	0.3354	0.6826	0.1065	0.1806	0.3953	0.7962
h_{33}	0.4506	0.4276	0.1784	0.58	0.4461	0.3586	0.2317
h_{34}	0.0441	0.0408	0.0298	0.0568	0.0453	-0.0114	0.0383
h_{35}	0.092	-0.0583	-0.1062	0.4247	0.2231	0.0744	-0.339
α_4	0.2365	0.1342	0.1031	0.1131	0.2284	0.1267	0.1571
g_{43}	0.5285	0.998	0.5437	-0.1823	0.6546	0.6093	-0.0593
g_{45}	0.0994	-0.1355	-0.3143	-0.0125	0.1155	-0.8466	-0.0963
β_4	2.0892	1.5964	1.0811	1.0991	1.8399	1.105	1.5641
h_{43}	-0.0075	0.0332	-0.3273	-0.8273	0.1598	-0.809	-0.7276
h_{44}	0.304	0.4755	0.5949	0.5315	0.2493	0.7565	0.5535
h_{45}	0.0484	-0.1392	-0.0778	0.1373	0.1064	-0.1407	-0.0017
α_5	1.406	1.2257	0.8236	0.7173	0.9558	1.1147	0.7605
g_{53}	0.2605	0.3664	0.4944	0.3046	0.4671	0.3687	0.3372
g_{54}	0.152	0.2235	0.2773	0.3722	0.3203	0.3649	0.345
g_{55}	0.0739	-0.1894	-0.3113	0.0463	-0.2207	-0.5318	-0.133
β_5	2.9437	2.1507	1.2926	0.771	1.1053	1.3387	0.9022
h_{51}	0.1962	0.255	0.3914	0.398	0.6407	0.1182	0.3734
h_{52}	0.1791	0.2845	0.2115	0.4764	0.4224	0.2067	0.2526
h_{53}	0.2354	0.0271	0.037	0.353	0.1648	-0.0016	0.3235
J_1		0.037	0.0227	0.023	0.018	0.0204	0.0207
J_2		0.0418	0.0582	0.0261	0.0188	0.0409	0.0865

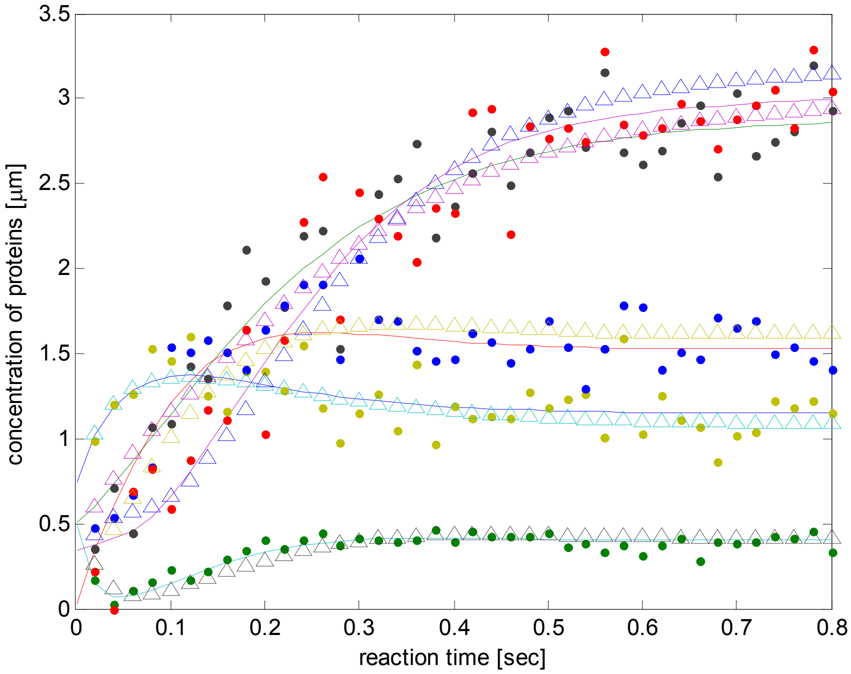

Fig. S11. The dynamic profiles of a trial with observation data subject to a noise level of 10% of standard deviation: solid lines represent “true” time-series data, dots represent the measured time-series data with added artificial noise, and triangles represent estimate time-series data produced by inferred model.

5. Gene network model

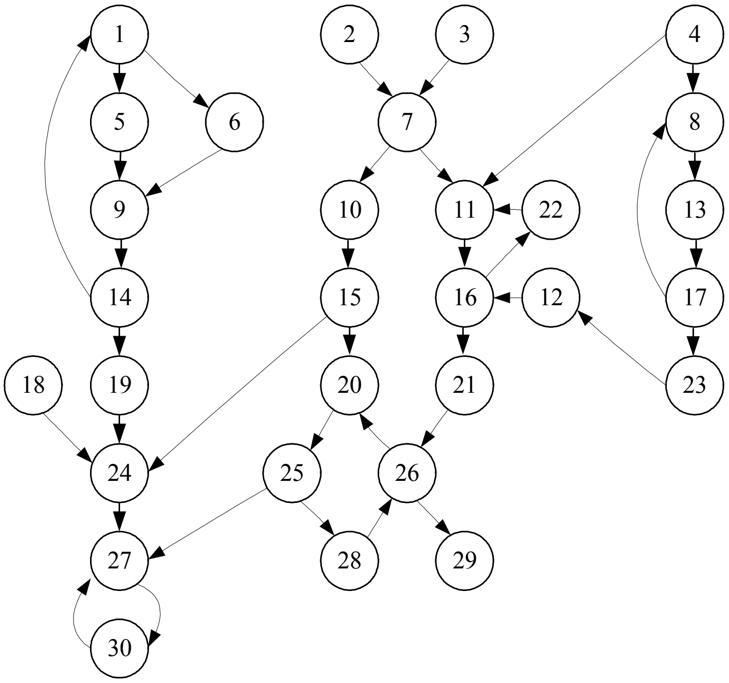

Fig. S11. Gene regulatory network composed of 30 genes

This genetic network contains 30 genes as shown in Fig. S11. The nominal rate constants and kinetic orders of the gene regulatory network model are given in the literature [13,14].

In [13], a cooperative co evolutionary is used to infer the genetic network. The computations were executed in parallel on a PC Cluster (Pentium III 933 MHz $\times$32 CPUs) and one experiment needs about 57.5 h [13]. Here, 5 sets of initial conditions in [0, 10.0] were generated. Each initial condition is then employed to generate the time-course data. The search ranges for each parameter used in the proposed spline-NLP method are αi and βi∈[0, 5], and gij and hij ∈[-3, 3]. We choice Simulation Annealing (SA) algorithm and the identification program is implemented on Matlab 7 on a single 2.13GHz CPU. The computational time required is 20 hr. To save space, we just give three trial results. The RSE J_1 and RPE J_2 are given in the last two row of each table, respectively in **Table S8-S10.**

Table S8. Nominal parameter values and experimental results of Parameter Estimation of the genetic network model in noise free condition

Nominal parameter value				Estimated parameters Noise level : 0%			
α_i	β_i	g_{ij}	h_{ij}	$\hat{\alpha}_i$	$\hat{\beta}_i$	$\hat{g}_{ij}$	$\hat{h}_{ij}$
1.00	1.00	-0.1	1.00	0.985	0.9817	-0.103	1.0221
1.00	1.00	1	1.00	1.6143	1.6241	0.9917	0.5293
1.00	1.00	1	1.00	0.9997	0.9997	0.9973	0.9997
1.00	1.00	0.5	1.00	1.4169	1.4428	0.4003	0.6986
1.00	1.00	0.4	1.00	1.0095	1.0092	0.3051	0.9892
1.00	1.00	0.2	1.00	1.0017	1.0018	0.1145	0.9955
1.00	1.00	-0.2	1.00	1.2121	1.213	-0.0961	0.8234
1.00	1.00	1	1.00	1.7201	1.74	0.8252	0.6153
1.00	1.00	-0.1	1.00	1.3436	1.3831	-0.1032	0.7372
1.00	1.00	0.3	1.00	1.7055	1.713	0.1787	0.5981
1.00	1.00	0.4	1.00	1.5015	1.5101	0.2369	0.7486
1.00	1.00	-0.2	1.00	1.2458	1.2937	-0.1054	0.7504
1.00	1.00	0.4	1.00	0.9629	0.9565	0.3444	1.0459
1.00	1.00	0.1	1.00	1.0008	1.0008	0.1055	0.9983

1.00	1.00	0.6	1.00	0.9994	0.9993	0.6179	1.0009
1.00	1.00	1	1.00	1.446	1.4442	0.9983	0.61
1.00	1.00	0.2	1.00	1.4648	1.4572	0.2009	0.7039
1.00	1.00	0.5	1.00	1.0742	1.0721	0.0906	0.889
1.00	1.00	-0.2	1.00	1.0045	1.0061	-0.0147	0.9933
1.00	1.00	0.5	1.00	1.5283	1.5197	0.3207	0.5914
1.00	1.00	0.1	1.00	1.2223	1.2304	0.0983	0.8179
1.00	1.00	0.7	1.00	1.514	1.5245	0.4296	0.6273
1.00	1.00	0.3	1.00	1.1501	1.1868	0.2066	0.8368
1.00	1.00	0.6	1.00	0.8634	0.8477	0.4604	1.1499
1.00	1.00	0.5	1.00	1.2766	1.2551	0.2874	0.6727
1.00	1.00	0.2	1.00	1.699	1.7157	0.2009	0.6039
1.00	1.00	-0.2	1.00	0.6187	0.6618	-0.2581	1.2787
1.00	1.00	-0.1	1.00	0.9308	0.9264	-0.0758	1.1026
1.00	1.00	0.3	1.00	1.0011	1.0011	0.3618	0.9988
1.00	1.00	0.4	1.00	1.6928	1.7003	0.296	0.6315
		-0.2				-0.1005	
		0.1				0.0768	
		0.6				0.3866	
		0.3				0.2736	
		-0.2				-0.0855	
		0.5				0.5572	
		0.4				0.3988	
		0.6				0.3243	
J_1				0.0009			
J_2				0.0013			

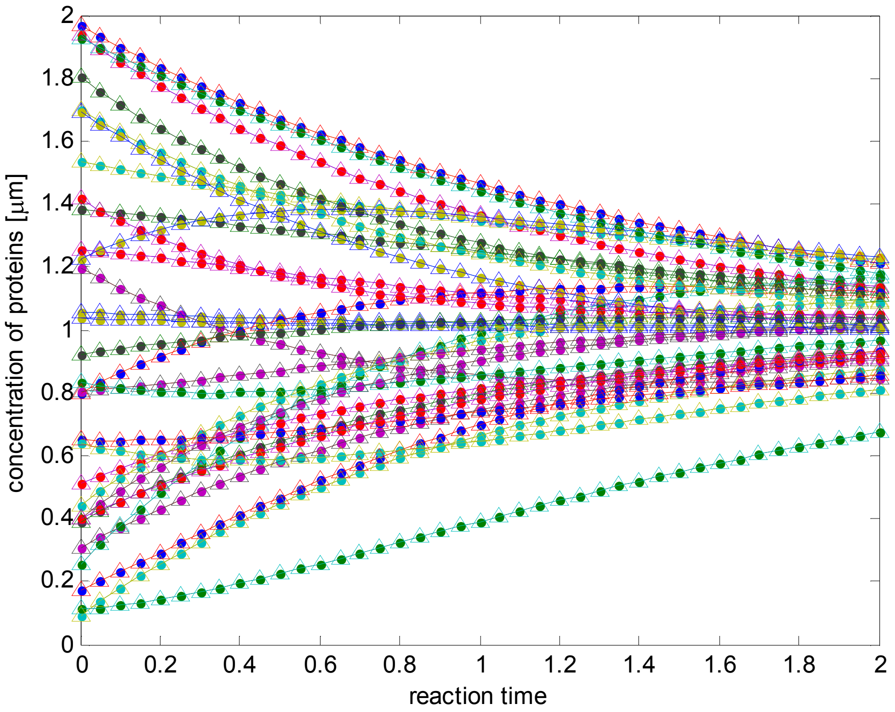

Fig. S12. The dynamic profiles of a trial in noise free condition: solid lines represent "true" time-series data, dots represent the measured time-series data with added artificial noise, and triangles represent estimate time-series data produced by inferred model.

Table S9. Nominal parameter values and experimental results of Parameter Estimation of the genetic network model with observation data subject to a noise level of 5% noise level condition

Nominal parameter value				Estimated parameters Noise level : 5%			
α_i	β_i	g_{ij}	h_{ij}	$\hat{\alpha}_i$	$\hat{\beta}_i$	$\hat{g}_{ij}$	$\hat{h}_{ij}$
1.00	1.00	-0.1	1.00	1.5542	1.4413	0.3363	0.9118
1.00	1.00	1	1.00	1.0123	0.9406	0.5639	0.7281
1.00	1.00	1	1.00	2.2769	2.1322	0.6601	0.5538
1.00	1.00	0.5	1.00	1.033	1.0666	0.2323	0.7572
1.00	1.00	0.4	1.00	1.3926	0.8113	1.5289	0.102
1.00	1.00	0.2	1.00	1.4271	1.616	-0.078	0.557
1.00	1.00	-0.2	1.00	1.3491	1.943	0.0823	0.8186
1.00	1.00	1	1.00	0.7509	0.8254	0.7959	1.0568
1.00	1.00	-0.1	1.00	1.7529	1.4225	-0.0253	0.6756
1.00	1.00	0.3	1.00	0.9852	0.8336	-0.4468	0.2792
1.00	1.00	0.4	1.00	1.1324	1.5115	0.1033	0.4745
1.00	1.00	-0.2	1.00	1.0923	1.0992	0.0406	0.2745
1.00	1.00	0.4	1.00	1.4103	1.2977	0.0118	1.0263
1.00	1.00	0.1	1.00	1.2902	1.4131	0.3939	0.9035
1.00	1.00	0.6	1.00	1.0885	1.0899	0.5834	0.8538

1.00	1.00	1	1.00	1.9354	2.0708	0.678	0.6417
1.00	1.00	0.2	1.00	1.1707	1.3296	-0.1967	0.493
1.00	1.00	0.5	1.00	1.4066	1.3356	0.3299	0.5612
1.00	1.00	-0.2	1.00	1.4102	1.2929	-0.057	0.3554
1.00	1.00	0.5	1.00	2.0218	2.0569	0.644	0.2446
1.00	1.00	0.1	1.00	1.1917	1.1072	0.1504	0.6987
1.00	1.00	0.7	1.00	1.9189	2.1142	0.5093	0.2631
1.00	1.00	0.3	1.00	1.3116	1.3084	0.3143	0.9272
1.00	1.00	0.6	1.00	1.1573	1.062	0.5666	0.8553
1.00	1.00	0.5	1.00	1.4854	1.4776	-0.0884	0.447
1.00	1.00	0.2	1.00	1.4724	1.6161	-0.0221	0.1911
1.00	1.00	-0.2	1.00	1.0602	1.0844	-0.0141	0.8927
1.00	1.00	-0.1	1.00	1.6519	1.6423	0.9595	0.4109
1.00	1.00	0.3	1.00	1.7244	1.8049	-0.0136	0.4488
1.00	1.00	0.4	1.00	1.7602	1.7277	0.1827	0.502
		-0.2				-0.7038	
		0.1				0.5616	
		0.6				0.5245	
		0.3				0.6156	
		-0.2				-0.0266	
		0.5				0.24	
		0.4				0.1724	
		0.6				0.3264	
J_1				0.0741			
J_2				0.0756			

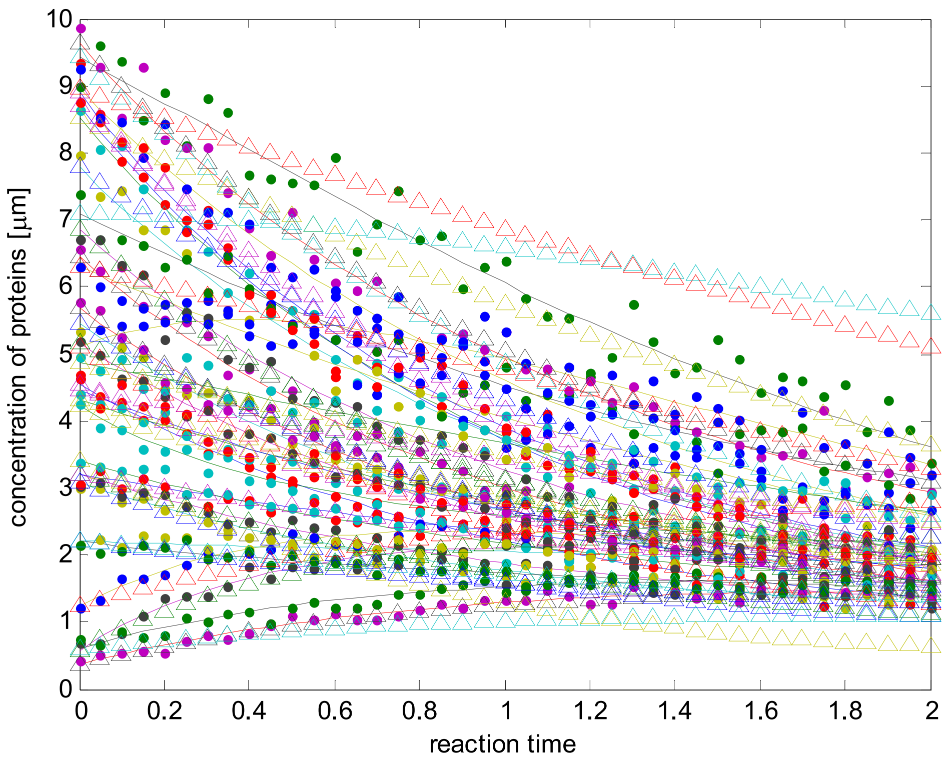

Fig. S13. The dynamic profiles of a trial with observation data subject to a noise level of 5% of standard deviation: solid lines represent "true" time-series data, dots represent the measured time-series data with added artificial noise, and triangles represent estimate time-series data produced by inferred model.

Table S10. Nominal parameter values and experimental results of Parameter Estimation of the genetic network model with observation data subject to a noise level of 10% noise level condition

Nominal parameter value				Estimated parameters Noise level : 10%			
α_i	β_i	g_{ij}	h_{ij}	$\hat{\alpha}_i$	$\hat{\beta}_i$	$\hat{g}_{ij}$	$\hat{h}_{ij}$
1.00	1.00	-0.1	1.00	1.2237	1.417	0.3219	0.8799
1.00	1.00	1	1.00	1.7187	2.1142	0.6081	0.3865
1.00	1.00	1	1.00	1.6346	1.767	0.9974	0.6758
1.00	1.00	0.5	1.00	1.4624	1.6223	-0.5499	0.7541
1.00	1.00	0.4	1.00	1.801	1.5995	0.3734	0.7008
1.00	1.00	0.2	1.00	0.6952	1.3851	0.1205	-0.191
1.00	1.00	-0.2	1.00	2.1377	1.3709	-0.1298	0.749
1.00	1.00	1	1.00	1.8935	1.9221	0.6378	0.6418
1.00	1.00	-0.1	1.00	1.2849	1.6019	-0.2791	0.4552
1.00	1.00	0.3	1.00	1.2962	0.9466	0.1509	1.0801
1.00	1.00	0.4	1.00	1.8117	1.9212	-0.3243	0.4548
1.00	1.00	-0.2	1.00	1.8447	2.056	-0.4958	0.5553
1.00	1.00	0.4	1.00	1.194	1.2418	0.4005	0.7511

1.00	1.00	0.1	1.00	2.0604	1.7411	0.0192	0.5318
1.00	1.00	0.6	1.00	1.3209	1.575	0.5018	0.7668
1.00	1.00	1	1.00	0.953	1.3501	0.6123	0.6639
1.00	1.00	0.2	1.00	1.7155	1.4309	0.2701	0.8426
1.00	1.00	0.5	1.00	1.9082	2.1171	0.1903	0.5629
1.00	1.00	-0.2	1.00	1.9327	1.428	0.3223	0.7905
1.00	1.00	0.5	1.00	1.7794	0.8756	0.2878	0.9156
1.00	1.00	0.1	1.00	1.0874	1.2425	-0.3885	0.4865
1.00	1.00	0.7	1.00	1.2928	1.4918	-0.4237	0.6942
1.00	1.00	0.3	1.00	1.8303	1.3901	-1.4487	0.7566
1.00	1.00	0.6	1.00	0.9363	1.0314	0.2784	1.0227
1.00	1.00	0.5	1.00	1.6547	1.5992	0.3804	0.5896
1.00	1.00	0.2	1.00	0.823	0.8068	-0.8269	0.9438
1.00	1.00	-0.2	1.00	1.2232	1.4719	-0.3024	0.3824
1.00	1.00	-0.1	1.00	1.6905	1.7429	0.7456	0.5348
1.00	1.00	0.3	1.00	0.7706	0.8316	-0.0924	1.107
1.00	1.00	0.4	1.00	0.9387	1.5104	0.0153	0.4692
		-0.2				-1.2085	
		0.1				-0.055	
		0.6				-0.3851	
		0.3				1.1324	
		-0.2				-0.1647	
		0.5				0.2786	
		0.4				0.9157	
		0.6				0.2626	
J_1				0.1008			
J_2				0.1449			

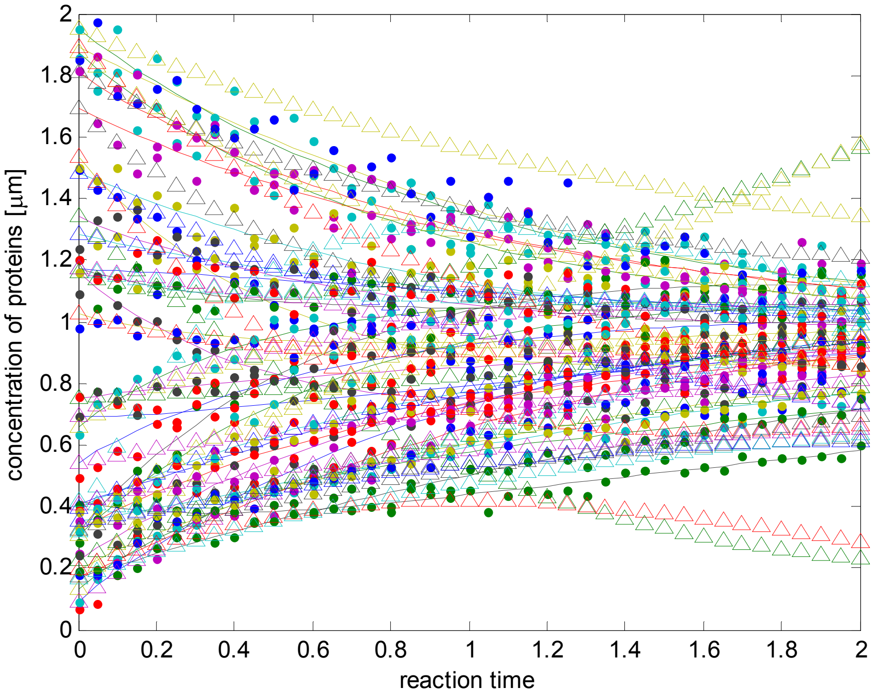

Fig. S14. The dynamic profiles of a trial with observation data subject to a noise level of 10% of standard deviation: solid lines represent "true" time-series data, dots represent the measured time-series data with added artificial noise, and triangles represent estimate time-series data produced by inferred model.

6. Three step pathway model

The optimization problem consists of the estimation of 36 kinetic parameters of a nonlinear biochemical dynamical model formed by 8 ODEs that describe the variation of the metabolite concentrations.

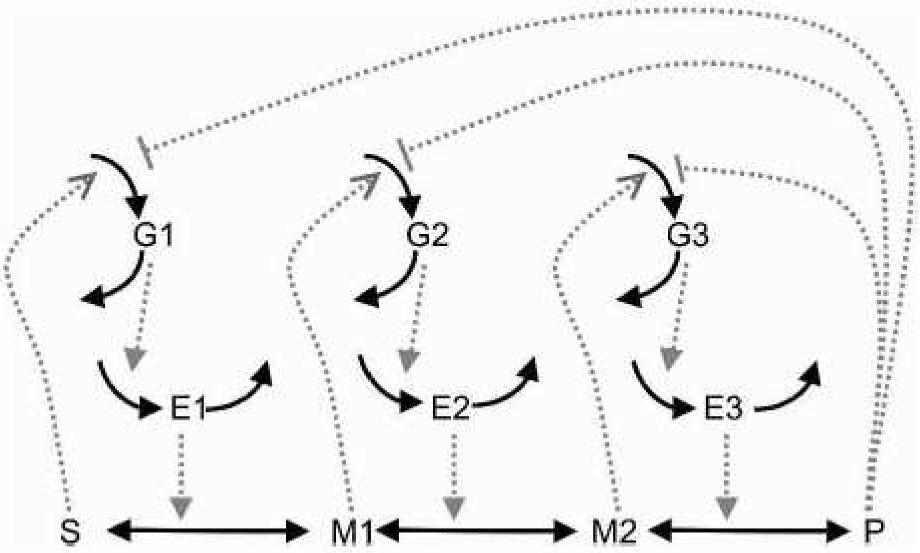

Fig. S.15 Graph representation of the three step model

The model metabolic pathway is used in these studies. The graph representation is shown in Fig. S.15. Solid arrows represent mass flow, dashed arrows represent kinetic regulation; arrow ends represent activation, blunt ends inhibition. S and P are the pathway substrate and product and are held at constant concentrations; M1 and M2 are intermediate metabolites of the pathway; E1, E2, and E3 are the enzymes; G1, G2, and G3 are the mRNA species for the enzymes.

Table S11. Definition of Variables for G1/S Transition Model

Symbol	x_1	x_2	x_3	x_4	x_5	x_6	x_7	x_8
Element	G_1	G_2	G_3	E_1	E_2	E_3	M_1	M_2

The mathematical formulation of the nonlinear dynamic model is:

$$\dot{x} = \frac{V_1 \cdot Ki_1^2 \cdot S^2}{Ki_1^2 \cdot S^2 + p^2 \cdot S^2 + Ka_1^2 \cdot Ki_1^2} - k_1 \cdot x_1$$

$$\dot{x}_2 = \frac{V_2 \cdot Ki_2^2 \cdot x_7^2}{Ki_2^2 \cdot x_7^2 + p^2 \cdot x_7^2 + Ka_2^2 \cdot Ki_2^2} - k_2 \cdot x_2$$

$$\dot{x}_3 = \frac{V_3 \cdot Ki_3^2 \cdot x_8^2}{Ki_3^2 \cdot x_8^2 + p^2 \cdot x_8^2 + Ka_3^2 \cdot Ki_3^2} - k_3 \cdot x_3$$

$$\dot{x}_4 = \frac{V_4 \cdot x_1}{K_4 + x_1} - k_4 \cdot x_4$$

$$\dot{x}_5 = \frac{V_5 \cdot x_2}{K_5 + x_2} - k_5 \cdot x_5$$

$$\dot{x}_6 = \frac{V_6 \cdot x_2}{K_6 + x_3} - k_6 \cdot x_6$$

$$\dot{x}_7 = \frac{Km_2 \cdot kcat_1 \cdot x_4 \cdot (S - x_7)}{Km_1 \cdot Km_2 + Km_2 \cdot S + Km_1 \cdot x_7} - \frac{Km_4 \cdot kcat_2 \cdot x_5 \cdot (x_7 - x_8)}{Km_3 \cdot Km_4 + Km_2 \cdot x_7 + Km_3 \cdot x_8}$$

$$\dot{x}_8 = \frac{Km_4 \cdot kcat_2 \cdot x_5 \cdot (x_7 - x_8)}{Km_3 \cdot Km_4 + Km_2 \cdot x_7 + Km_3 \cdot x_8} - \frac{Km_6 \cdot kcat_3 \cdot x_6 \cdot (x_8 - p)}{Km_3 \cdot Km_6 + Km_6 \cdot x_8 + Km_5 \cdot p}$$

Where x_1, x_2, x_3 x_4, x_5, x_6, x_7, and x_8 represent the concentrations of the species involved in the different biochemical reactions and S and P keep fixed initial values for each experiment.

In [12], many parameter estimation approaches are compared and the best result are achieved after 39.42 h computation time. Here, the Stochastic Raking Evolution Strategy (SRES) algorithm is selected to implement the optimization [15]. SRES uses stochastic ranking as the constraint handling technique, which adjusts the balance between the objective and penalty functions automatically during the evolutionary

search. For trials with noise free data, the algorithm converges in 4 hours after 80000 iterations. However, this large computational effort was the consequence of the very tight convergence criteria, but from a practical point of view, an almost equal good result was reached in about 60000 generations. Results of just a few selected trials are shown in Table S12-S14.

In high noise condition, we found that the estimated results of km_2, $kcat_2$, km_3, km_4, $kcat_3$ and km_5 are always far from their nominal value, but the identified system still produces satisfactory system responses (shown in Table S13 and Fig. S16). This phenomenon implies that the system may not sensitive with these parameters and the system may be sloppy system. We try to increase the generations of the algorithm, but the improvement of the estimation results is not obvious.

Table S12: Some experimental Results of Parameter Estimation of the mammalian three step pathway model in noise free condition

Parameters	True value	Estimated parameters Noise level : 0%					
V_1	1	0.9997	0.9995	0.9995	0.9995	0.9996	0.9995
Ki_1	1	1	0.9999	0.9999	0.9999	1	0.9999
Ka_1	1	1	1	1	1	1	1
k_1	1	0.9997	0.9995	0.9995	0.9995	0.9996	0.9995
V_2	1	0.9997	0.9999	0.9999	0.9999	0.9997	0.9999
Ki_2	1	1	1.0001	1	1	1	1
ka_2	1	1	0.9999	0.9999	0.9999	1	0.9999
k_2	1	0.9997	0.9999	0.9999	0.9999	0.9997	0.9999
V_3	1	0.9997	0.9993	0.9993	0.9994	0.9997	0.9994
Ki_3	1	1	1.0004	1.0004	1.0003	1.0002	1.0003
Ka_3	1	0.9999	0.9998	0.9998	0.9998	0.9999	0.9998
k_3	1	0.9997	0.9995	0.9995	0.9995	0.9998	0.9995
V_4	0.1	0.1	0.1	0.1	0.1	0.1	0.1
K_4	1	1.0004	0.9998	0.9997	0.9998	1	0.9998

k_4	0.1	0.1	0.1	0.1	0.1	0.1	0.1
V_5	0.1	0.1	0.1	0.1	0.1	0.1	0.1
K_5	1	1.0007	1.0002	1.0002	1.0002	1.0006	1.0002
k_5	0.1	0.1	0.1	0.1	0.1	0.1	0.1
V_6	0.1	0.1	0.1	0.1	0.1	0.1	0.1
K_6	1	1.0002	1.0003	1.0003	1.0003	0.9999	1.0003
k_6	0.1	0.1	0.1	0.1	0.1	0.1	0.1
$kcat_1$	1	1.0001	1	1	1	1	1
km_1	1	1.0002	0.9999	1	1	1	1
km_2	1	1.0003	1	0.9999	0.9999	0.9999	0.9999
$kcat_2$	1	1.0007	1	1	1	0.9999	1
km_3	1	1.0008	0.9999	0.9999	0.9999	1	0.9999
km_4	1	1	0.9999	0.9998	0.9998	1	0.9998
$kcat_3$	1	1.0006	1	0.9999	0.9999	1	0.9999
km_5	1	0.9369	1.135	1.9639	1.6872	8.1043	4.6079
km_6	1	0.9363	1.135	1.9641	1.6873	8.1035	4.6081
J_1		0	0	0	0	0	0
J_2		0	0	0	0	0	0

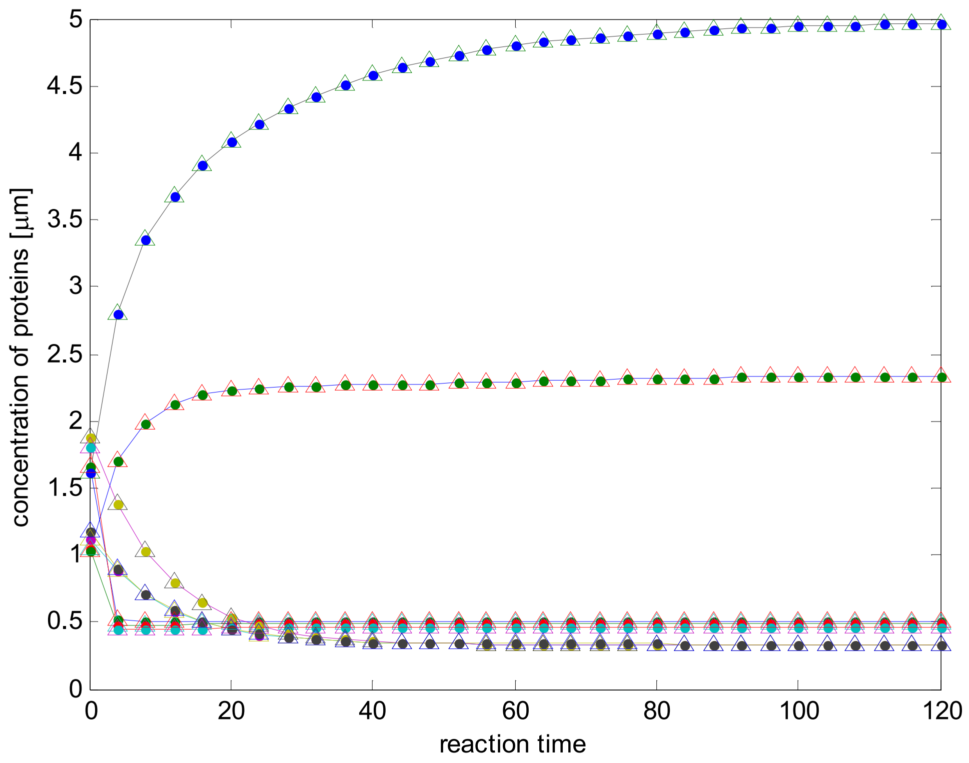

Fig. S15. The dynamic profiles of a trial with observation data subject to a noise level of 0% of standard deviation: solid lines represent "true" time-series data, dots represent the measured time-series data with added artificial noise, and triangles represent estimate time-series data produced by inferred model.

Table S.12: Some experimental Results of Parameter Estimation of three step pathway model with 5% noise

Parameters	True value	Estimated parameters Noise level : 5%					
V_1	1	1.0273	1.032	0.9474	1.0318	0.9894	0.9932
Ki_1	1	0.966	0.9822	1.2104	0.9493	0.9955	1.079
Ka_1	1	1.0878	0.9733	0.9461	1.0139	1.0115	1.0354
k_1	1	0.9877	1.0323	0.9929	0.9966	0.9957	0.9832
V_2	1	1.0904	0.9698	1.0933	1.0514	0.9277	1.0383
Ki_2	1	0.9854	0.9886	0.9485	0.9808	1.0478	0.9705
ka_2	1	1.0102	1.0086	1.0281	1.0428	1.0177	1.0138
k_2	1	1.0281	0.9761	1.0258	1.003	0.9648	0.9967
V_3	1	0.9993	0.8411	0.864	1.0064	0.9977	0.9354

Ki_3	1	0.9897	1.0522	1.0375	0.9685	0.9786	1.0601
Ka_3	1	1.022	0.9685	0.9916	1.0289	0.9957	1.0024
k_3	1	0.9946	0.9459	0.9534	0.9718	1.0155	1.011
V_4	0.1	0.2625	0.178	0.4008	0.3977	0.2091	0.2771
K_4	1	5.0662	2.7586	8.2346	9.714	3.6635	5.7162
k_4	0.1	0.0959	0.0938	0.0958	0.0926	0.0942	0.083
V_5	0.1	2.8863	5.141	4.4559	3.777	3.6101	4.3512
K_5	1	79.1256	83.6937	74.972	91.762	70.1185	83.7462
k_5	0.1	0.0884	0.1001	0.1001	0.0901	0.095	0.095
V_6	0.1	0.4742	0.6309	0.7548	0.3265	0.5963	0.6553
K_6	1	8.7108	7.692	9.6906	4.4695	7.7345	8.5135
k_6	0.1	0.0872	0.1072	0.102	0.0993	0.0978	0.1034
$kcat_1$	1	0.8685	0.8807	0.9017	0.912	0.8484	0.9101
km_1	1	1.7974	1.5517	1.3885	1.9548	1.8166	1.129
km_2	1	49.5427	4.8053	3.2977	77.1399	57.4746	1.5398
$kcat_2$	1	41.1747	0.8925	5.9677	25.1306	46.8873	0.8619
km_3	1	85.7832	1.661	10.2108	54.3206	96.8373	1.2097
km_4	1	2.118	5.2283	1.1876	3.9829	1.9286	1.9962
$kcat_3$	1	48.1231	1.2614	6.2047	31.8923	56.062	1.0766
km_5	1	5.8687	9.732	4.3048	9.3694	9.2161	8.2494
km_6	1	0.0473	7.1356	0.2119	0.1112	0.068	7.5893
J_1		0.0019	0.0009	0.0017	0.0026	0.003	0.0025
J_2		0.0154	0.0028	0.0075	0.0178	0.0081	0.0056

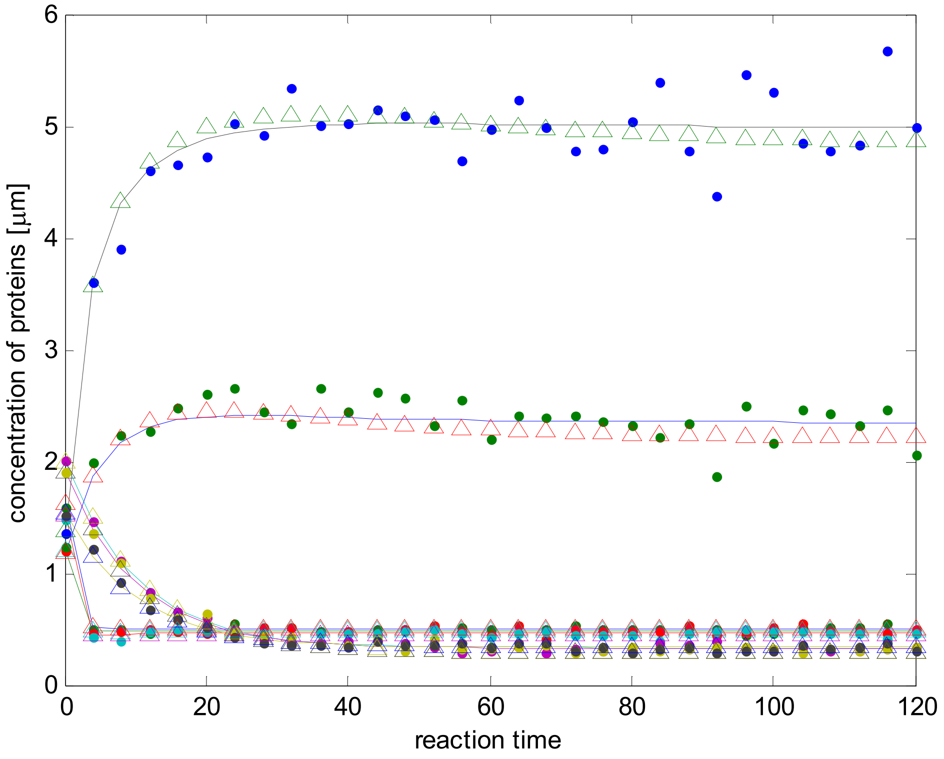

Fig. S16. The dynamic profiles of a trial with observation data with 5% noise: solid lines represent "true" time-series data, dots represent the measured time-series data with added artificial noise, and triangles represent estimate time-series data produced by inferred model.

Table S.13: Some experimental Results of Parameter Estimation of three step pathway model with 10% noise

Parameters	True value	Estimated parameters Noise level : 10%					
V_1	1	0.9605	1.0069	1.0659	0.9681	0.9076	0.924
Ki_1	1	1.1927	1.3267	0.9058	0.9169	1.5884	1.0752
Ka_1	1	0.8493	1.1282	0.9584	0.9679	1.068	0.8286
k_1	1	1.0601	0.9992	0.9972	0.9777	0.9658	1.0091
V_2	1	0.8439	0.9544	0.9259	0.8503	0.7413	1.0732
Ki_2	1	1.044	1.0669	0.9574	1.0172	1.1493	0.94
ka_2	1	1.0236	1.1691	1.1201	1.0048	0.9005	1.0225
k_2	1	0.9479	0.919	0.9366	0.9054	0.9134	1.0213
V_3	1	1.0866	0.6336	0.9691	0.7893	7.7428	0.505

Ki_3	1	0.8437	1.2612	1.0485	1.0129	0.2648	6.7387
Ka_3	1	1.1559	0.8595	1.2209	0.8997	3.2879	0.7684
k_3	1	0.9362	0.8784	0.8563	0.9468	0.9521	0.9047
V_4	0.1	0.262	0.2992	0.4421	0.7876	0.5246	0.431
K_4	1	5.7622	2.4484	8.1834	9.7793	6.9212	6.9818
k_4	0.1	0.0946	0.1104	0.1043	0.113	0.1189	0.0825
V_5	0.1	4.7494	4.3025	1.7759	2.2051	6.6367	2.4411
K_5	1	48.626	93.3281	67.3261	71.4966	94.2742	54.6746
k_5	0.1	0.1002	0.1032	0.0858	0.0898	0.1016	0.0944
V_6	0.1	0.5968	0.6055	0.4413	0.5483	0.6581	0.3871
K_6	1	8.6853	7.1564	6.5852	6.1167	8.19	9.038
k_6	0.1	0.1024	0.1057	0.1019	0.1015	0.096	0.0825
$kcat_1$	1	0.8211	90.078	0.921	0.7734	0.8278	1.0935
km_1	1	1.6844	52.5402	2.8994	1.9777	1.4091	3.0174
km_2	1	85.31	0.1647	79.4996	84.5769	29.9077	55.402
$kcat_2$	1	14.8522	23.9172	20.895	27.7786	39.1293	34.7247
km_3	1	46.3033	95.9871	85.2208	92.8295	77.0596	94.304
km_4	1	4.9476	83.6203	54.3254	10.9675	1.21	47.9378
$kcat_3$	1	21.487	50.2946	43.0069	46.8058	38.9805	49.7799
km_5	1	5.2768	9.8334	5.6081	6.2579	1.26	0.1785
km_6	1	0.0977	0.1843	0.084	0.055	7.3858	1.87
J_1		0.009	0.0041	0.0048	0.0053	0.0106	0.0129
J_2		0.0207	0.0366	0.0332	0.0254	0.0155	0.0116

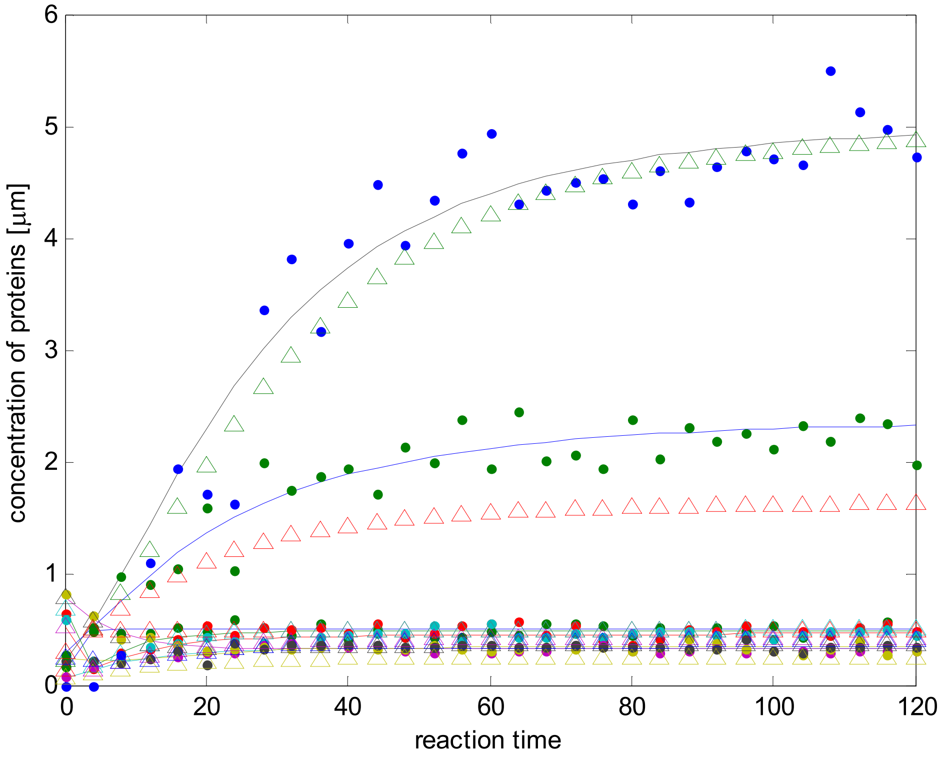

Fig. S17. The dynamic profiles of a trial with observation data subject to a noise level of 10% of standard deviation: solid lines represent “true” time-series data, dots represent the measured time-series data with added artificial noise, and triangles represent estimate time-series data produced by inferred model.

7. More experimental results of the mammalian G1/S transition network model (Swat et al., 2004)

The graph representation of TNFα-Mediated NF-κB-Signalling Pathway Model is shown in Fig. S18. More details can be found in [11]

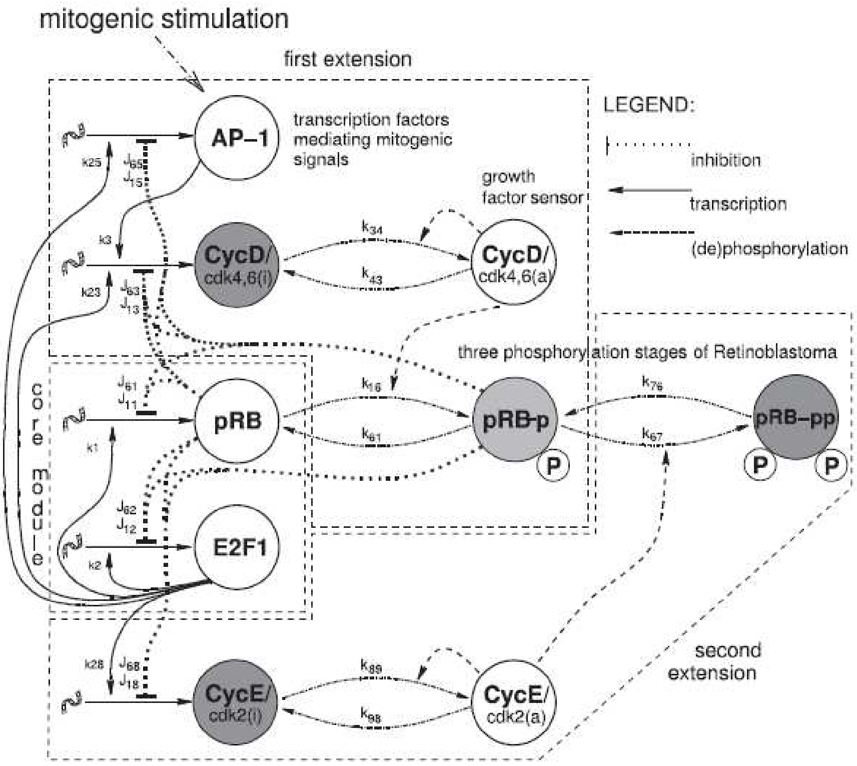

Fig. S18 Graphical representation of the mammalian G1/S transition network model:

The details of experiments are given in the Section 4 of the Paper. Hence, it will not be repeated again here. We just show more Results of trials in Table S12-S14.

Table S.14: Some experimental Results of Parameter Estimation of the mammalian G1/S transition network model in noise free condition

Parameters	True value	Estimated parameters Noise level : 0%			
k_1	1	0.9675	0.9933	0.9957	1.0095
k_2	1.6	1.6047	1.598	1.5989	1.6001
k_3	0.05	0.05	0.0498	0.05	0.05
k_{16}	0.4	0.3993	0.3999	0.4002	0.4002
k_{34}	0.04	0.0399	0.0401	0.04	0.04
k_{43}	0.01	0.0097	0.0095	0.01	0.01
k_{61}	0.3	0.2894	0.2988	0.2985	0.3008
k_{67}	0.7	0.6994	0.7003	0.6999	0.6999

k_{76}	0.1	0.0999	0.1	0.0999	0.1
k_{23}	0.3	0.5437	0.1355	0.1219	1.0665
k_{25}	0.9	0.0427	1.542	0.1785	2.5624
k_{28}	0.06	0.0601	0.06	0.0601	0.06
k_{89}	0.07	0.07	0.07	0.07	0.07
k_{98}	0.01	0.0098	0.01	0.01	0.01
a	0.04	0.1273	0.03	0	0.0007
J_{11}	0.5	0.5202	0.5042	0.4992	0.4989
J_{12}	5	5.0063	5.0142	5.0025	4.9959
J_{15}	0.001	0.0209	0.0006	0.0051	0.0004
J_{18}	0.6	0.6019	0.5986	0.599	0.6003
J_{61}	5	6.3317	5.1641	5.2581	4.8024
J_{62}	8	7.9551	8.0209	8.0088	8.0004
J_{65}	6	9.4936	8.7068	5.9222	6.0152
J_{68}	7	6.8859	7.0054	6.9916	6.9994
J_{13}	0.002	0.0008	0.0036	0.005	0.0006
J_{63}	2	25.8081	173.121	1.974	1.9983
K_{m1}	0.5	0.4912	0.4989	0.4905	0.5032
K_{m2}	4	4.004	3.9961	3.9985	16.0003
K_{m4}	0.3	0.2978	0.3077	0.2999	0.3
K_{m9}	0.005	0.0036	0.0045	0.0054	0.005
k_p	0.05	0.0496	0.0496	0.0499	0.0501
ϕ_1	0.005	0.0045	0.0048	0.0044	0.0053
ϕ_2	0.1	0.1002	0.0999	0.0999	0.1
ϕ_3	0.023	0.0228	0.0226	0.023	0.023
ϕ_4	0.03	0.0302	0.0306	0.03	0.03
ϕ_5	0.01	0.01	0.01	0.01	0.01
ϕ_6	0.06	0.0693	0.0609	0.0606	0.0593

ϕ_7	0.04	0.0399	0.04	0.0401	0.04
ϕ_8	0.06	0.0599	0.0599	0.06	0.06
ϕ_9	0.05	0.0502	0.05	0.05	0.05
J_1		0.0001	0.0002	0	0.0025
J_2		0.0001	0.0002	0	0.0082

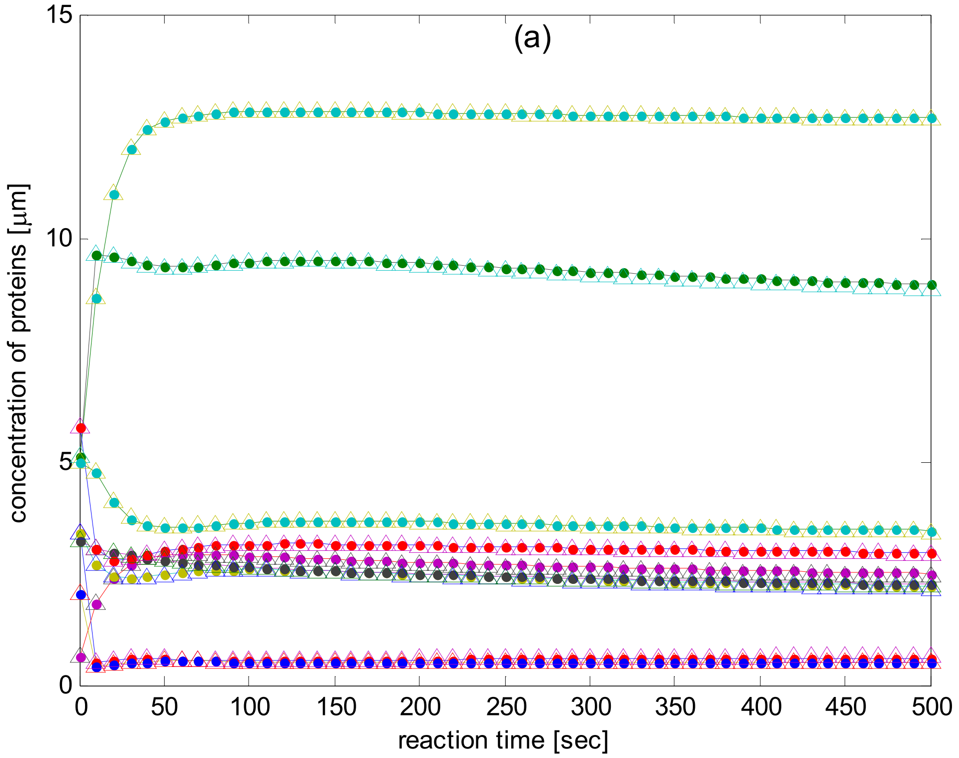

Fig. S19. The dynamic profiles of a trial with observation data in noise free condtion: solid lines represent "true" time-series data, dots represent the measured time-series data with added artificial noise, and triangles represent estimate time-series data produced by inferred model.

Table S.15: Some experimental Results of Parameter Estimation of the mammalian G1/S transition network model with 5% noise

Parameters	True value	Estimated parameters Noise level : 5%			
k_1	1	0.5329	0.4835	0.5879	0.5054
k_2	1.6	1.2836	5.0423	2.7994	1.1479
k_3	0.05	0.0642	0.0567	0.0601	0.0403

k_{16}	0.4	0.2968	0.3137	0.331	0.3227
k_{34}	0.04	0.0435	0.0279	0.0587	0.037
k_{43}	0.01	0.0164	0.011	0.0294	0.0138
k_{61}	0.3	0.1851	0.243	0.0611	0.2525
k_{67}	0.7	0.5355	0.5062	0.6666	0.5314
k_{76}	0.1	0.0769	0.064	0.0986	0.0764
k_{23}	0.3	1.6503	0.0937	0.1286	0.277
k_{25}	0.9	7.7194	0.2992	0.0351	2.3029
k_{28}	0.06	0.0345	0.4058	0.0307	0.0369
k_{89}	0.07	0.0662	0.077	0.0696	0.0692
k_{98}	0.01	0.0051	0.0295	0.0218	0.0048
a	0.04	0.0598	0.0694	0.0251	0.0184
J_{11}	0.5	5.2456	9.1093	6.6892	4.4941
J_{12}	5	139.4297	19.3794	52.8352	151.1956
J_{15}	0.001	0	0.0026	0.0353	0.0002
J_{18}	0.6	5.5692	0.0316	24.9904	1.1877
J_{61}	5	14.1477	26.0591	25.8425	236.706
J_{62}	8	12.2039	43.2532	67.293	293.9981
J_{65}	6	33.9126	17.9855	15.3993	23.0246
J_{68}	7	6.7511	39.3314	40.7034	24.5825
J_{13}	0.002	0.0012	0.0018	0.0117	0.0003
J_{63}	2	4.4223	19.3614	0.0117	15.3326
K_{m1}	0.5	0.458	2.7802	0.6866	0.2282
K_{m2}	4	4.0870	9.1343	7.1589	3.2915
K_{m4}	0.3	0.8519	0.044	1.1157	0.2822
K_{m9}	0.005	0.1462	0.0756	0.0947	0.1403
k_p	0.05	0.0285	0.7393	0.3961	0.0227
ϕ_1	0.005	0.1512	0.0854	0.1403	0.2299

ϕ_2	0.1	0.0853	0.3145	0.1946	0.0811
ϕ_3	0.023	0.0473	0.0383	0.0346	0.0242
ϕ_4	0.03	0.0193	0.0196	0.0264	0.0278
ϕ_5	0.01	0.0079	0.0102	0.0147	0.0071
ϕ_6	0.06	0.0716	0.0125	0.1727	0.0355
ϕ_7	0.04	0.0313	0.0351	0.0361	0.0318
ϕ_8	0.06	0.0705	0.0599	0.0849	0.0372
ϕ_9	0.05	0.0466	0.037	0.034	0.0497
J		0.0441	0.0306	0.0408	0.0212

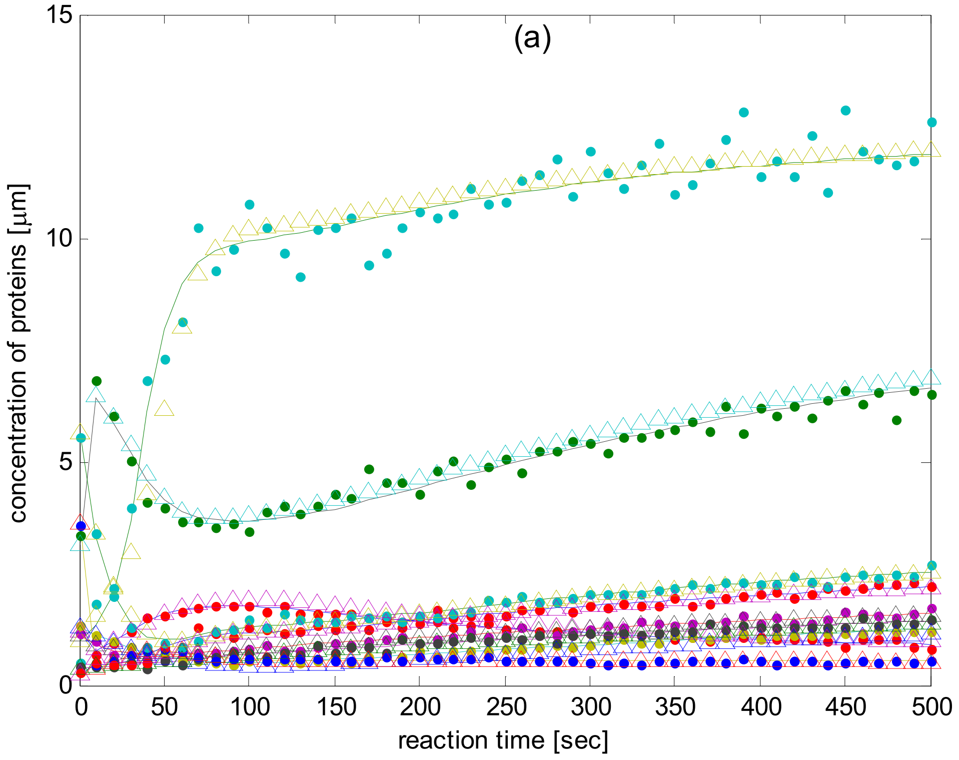

Fig. S20. The dynamic profiles of a trial with observation data subject to a noise level of 5% of standard deviation: solid lines represent "true" time-series data, dots represent the measured time-series data with added artificial noise, and triangles represent estimate time-series data produced by inferred model.

Table S.16: Some experimental Results of Parameter Estimation of the mammalian G1/S transition network model with 10% noise condition

Parameters	True value	Estimated parameters Noise level : 10 %			
k_1	1	0.6226	0.48	0.6192	0.2821
k_2	1.6	1.4415	1.508	1.4015	2.6773
k_3	0.05	0.0756	0.0372	0.0344	0.0244
k_{16}	0.4	0.2163	0.3357	0.2994	0.1595
k_{34}	0.04	0.0136	0.1319	0.1054	0.0257
k_{43}	0.01	0.0013	0.0058	0.0095	0.0018
k_{61}	0.3	0.1287	0.2096	0.104	0.0676
k_{67}	0.7	0.3329	0.5077	0.6007	0.3753
k_{76}	0.1	0.0378	0.064	0.0777	0.0562
k_{23}	0.3	0.0441	0.3355	2.5651	0.0107
k_{25}	0.9	0.6984	1.2132	0.2062	0.967
k_{28}	0.06	0.0938	0.0815	0.0191	0.0285
k_{89}	0.07	0.0945	0.0839	0.0627	0.0554
k_{98}	0.01	0.001	0.0158	0.0025	0.0004
a	0.04	0.0917	0.04	0.024	0.0787
J_{11}	0.5	0.7252	17.6315	14.183	1.4671
J_{12}	5	10.0118	93.5043	13.5551	4.8565
J_{15}	0.001	0.0016	0.0005	0.0047	0.0016
J_{18}	0.6	0.2427	0.3172	11.1283	1.0144
J_{61}	5	4.8894	26.0577	19.6182	5.3156
J_{62}	8	28.2461	23.3613	20.5402	26.3265
J_{65}	6	5.3094	42.4219	48.5328	5.9955
J_{68}	7	4.6726	8.2274	38.6839	13.3383
J_{13}	0.002	0.0002	0.006	0.0022	0.0001
J_{63}	2	3.6913	12.2252	18.5189	0.5441
K_{m1}	0.5	2.7564	0.6586	1.2336	0.1367
K_{m2}	4	6.6045	28.0248	17.2677	7.053

K_{m4}	0.3	0.1192	8.5114	9.0768	0.3702
K_{m9}	0.005	0.0293	0.0597	0.1434	0.0298
k_p	0.05	0.1505	0.1083	0.056	0.2046
ϕ_1	0.005	0.0106	0.1012	0.0741	0.0174
ϕ_2	0.1	0.094	0.1139	0.11	0.1551
ϕ_3	0.023	0.0553	0.0138	0.0383	0.0021
ϕ_4	0.03	0.013	0.022	0.0118	0.0231
ϕ_5	0.01	0.0119	0.0094	0.0054	0.0157
ϕ_6	0.06	0.0237	0.0445	0.0766	0.0475
ϕ_7	0.04	0.0257	0.0433	0.0451	0.0169
ϕ_8	0.06	0.0049	0.0534	0.0593	0.0242
ϕ_9	0.05	0.0796	0.06	0.0491	0.0462
J_1		0.0103	0.1048	0.1963	0.0087

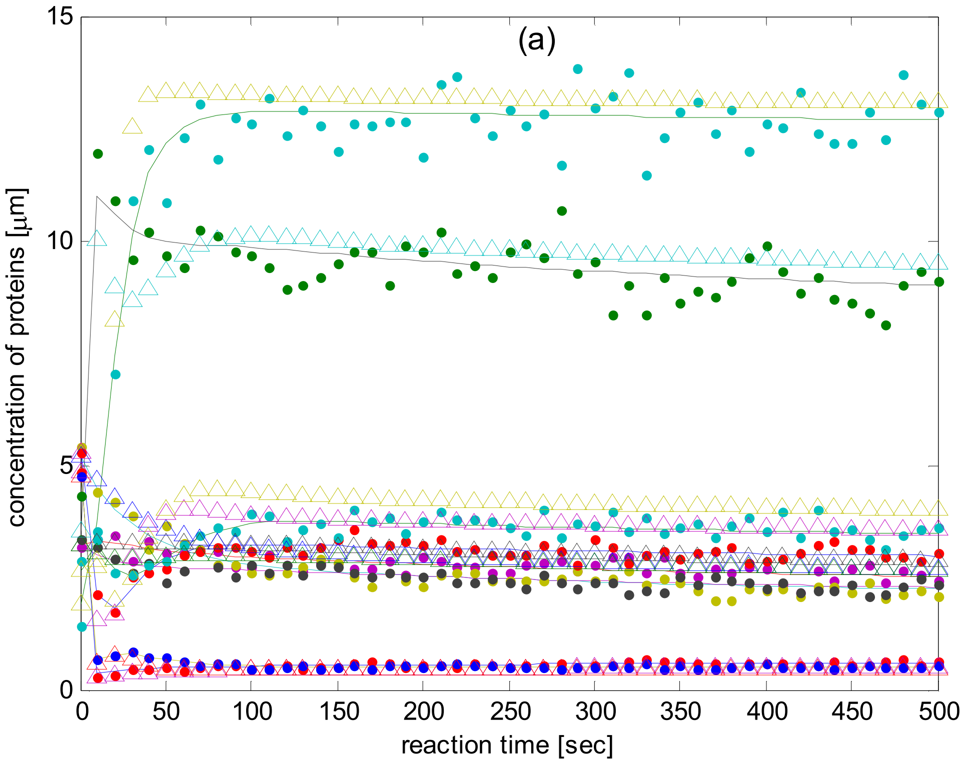

Fig. S21. The dynamic profiles of a trial with observation data subject to a noise level of 10% of standard deviation: solid lines represent "true" time-series data, dots

represent the measured time-series data with added artificial noise, and triangles represent estimate time-series data produced by inferred model.

Reference

1. Cho KH., Shin SY., Lee HW., and Wolkenhauer O., “Investigations in the Analysis and Modelling of the TNFalpha Mediated NF-kappaB Signaling Pathway,” Genome Research, vol. 13, pp. 2413-2422, 2003.
2. Cho KH., Shin SY., Kim HW., Wolkenhauer O., McFerran B., and Kolch W. Mathematical modeling of the influence of RKIP on the ERK signaling pathway, Computational Methods in Systems Biology (CMSB’03). vol. 2602 of Lecture Notes in Computer Science., Springer-Verlag, 2003.
3. Lall R, Voit EO. (2005) “Parameter estimation in modulated, unbranched reaction chains within biochemical systems”, Comput Biol Chem. 29(5): 309-18.
4. Polisetty PK, Voit EO, Gatzke EP. (2006) “Identification of metabolic system parameters using global optimization methods”, Theor Biol Med Model. 27;3:4.
5. Chou IC, Martens H, Voit EO. (2006) “Parameter estimation in biochemical systems models with alternating regression”, Theor Biol Med Model. 19;3:25.
6. Koh G, Teong HF, Clément MV, Hsu D, Thiagarajan PS. (2006) “A decompositional approach to parameter estimation in pathway modeling: a case study of the Akt and MAPK pathways and their crosstalk”, Bioinformatics, 22 (24), pp: 271-280.
7. Kuzmic, P., Program DYNAFIT for the Analysis of Enzyme Kinetic Data: Application to HIV Proteinase. 1996, Anal. Biochem., 237, 260-273.
8. Mendes P, Kell D. (1998) “Non-linear optimization of biochemical pathways: application to metabolic engineering and parameter estimation”, Bioinformatics. 14(10):869-83.
9. Cho DY, Cho KH, Zhang BT. (2006) “Identification of biochemical networks by S-tree based genetic programming”, Bioinformatics, 22(13), pp:1631-1640.

10. Vera J, de Atauri P, Cascante M, Torres NV. (2003) Multicriteria optimization of biochemical systems by linear programming: Application to production of ethanol by Saccharomyces cerevisiae. Biotechnol., Bioeng., 83, 335-343.
11. Swat M, Kel A, Herzel H., "Bifurcation analysis of the regulatory modules of the mammalian G_1/S transition", Bioinformatics. 2004, 20(10):1506-1511.
12. Moles CG, Mendes P, Banga JR. (2003) "Parameter estimation in biochemical pathways: a comparison of global optimization methods", Genome Res. 13(11):2467-74.
13. Kimura S, Ide K, Kashihara A, Kano M, Hatakeyama M, Masui R, Nakagawa N, Yokoyama S, Kuramitsu S, Konagaya A. (2005) Inference of S-system models of genetic networks using a cooperative coevolutionary algorithm, Bioinformatics. 21(7):1154-63.
14. Maki, Y., Tominaga, D, Okamoto, Watanabe, S. and Eguchi, Y. (2001) Development of a system for the inference of large scale genetic netoworks. Proc. Pac. Symp. Biocomput., 6, 446-458.
15. Runarsson TP, Yao X., 2000. Stochastic ranking for constrained evolutionary optimization. IEEE Trans. Evol. Comput. 4: 284-294.
16. Kimura, S., Sonoda, K., Yamane, S., Matsumura, K., and Hatakeyama, M. (2007) Function approximation approach to the inference of neural network models of genetic networks. IPSJ Digital Courier, 3, 153-163
