# Supplementary material for: Parameter estimation in systems biology models using spline approximation
Source: BMC Syst Biol. 2011 Jan 24;5:14. doi: 10.1186/1752-0509-5-14 (PMC3750107; doi:10.1186/1752-0509-5-14)
Supplement: Additional file 2 — In this additional file, we use E2F/DP dimmer model to illustrate the differences between the three different type methods mentioned in the paper: (i) the direct optimization method; (ii) decomposition methods; (iii) methods Combine spline theory and NLP. [file 1752-0509-5-14-S2.PDF]

Supplementary 2： Analysis of different approaches

Let us consider the following E2F/DP dimmer model [4],

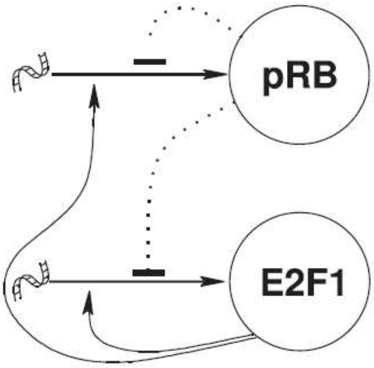

Fig. S1 The double inhibitor–activator module

The ODEs model is shown as following

$$\frac{d[pRB]}{dt} = k_1 \frac{[E2F1]}{K_{m1} + [E2F1]} \frac{J_{11}}{J_{11} + [pRB]} - \phi_{pRB}[pRB]$$
$$\frac{d[E2F1]}{dt} = k_p + k_2 \frac{a^2 + [E2F1]^2}{K_{m2}^2 + [E2F1]^2} \frac{J_{12}}{J_{12} + [pRB]} - \phi_{E2F1}[E2F1] \quad (1)$$

The parameter values are shown in Table S1. Figure S2 is a dynamic profile of the system with noise free data.

Table S1. Parameter values

k_1	K_{m1}	J_{11}	ϕ_{pRB}	k_p	k_2	a	K_{m2}	J_{12}	ϕ_{E2F1}
1	0.5	0.5	0.005	0.05	1.6	0.04	4	5	0.1

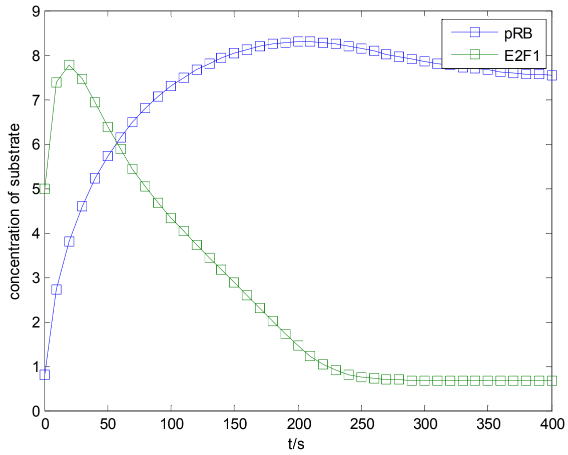

Fig. S2 The dynamic profile of a trial with observation data subject to 0% noise

S1 The direct optimization method or Single shooting method

The parameter estimation problem can be formulated as a nonlinear programming problem (NLP) with differential-algebraic constraints, which finds a set of optimal parameters $\hat{\theta} \in R^k$, so that the responses from the ODEs model is in best alignment with the measurement data:

$$P_0: \min_{\hat{\theta},\hat{x}_0} \sum_{j=0}^{N-1} (y(t_j) - \hat{y}(t_j|\hat{\theta}))^T W_j (y(t_j) - \hat{y}(t_j|\hat{\theta})),$$

$$s.t. \begin{cases} \text{(i)} \quad \dot{\hat{x}}(t_j) = f(\hat{x}(t_j|\hat{\theta}), u(t), \hat{\theta}), \quad x(t_0) = \hat{x}_0, \\ \text{(ii)} \quad \hat{y}(t_j) = g(\hat{x}(t_j|\hat{\theta})), \quad j = 1, 2, \cdots, N-1, \\ \text{(iii)} \quad C_{eq}(\dot{\hat{x}}, \hat{x}, \hat{\theta}) = 0, \\ \text{(iv)} \quad C_{ineq}(\dot{\hat{x}}, \hat{x}, \hat{\theta}) \le 0, \\ \text{(v)} \quad \theta_L \le \hat{\theta} \le \theta_U . \end{cases} \tag{2}$$

Here, P_0 minimizes a cost function that measures the fitness of the model with respect to a given set of experiment data subjecting to a set of constraints, where $\hat{\theta} \in R^k$ is the set of parameters to be estimated, $\hat{x}_0$ is the estimated initial condition, $\hat{x} \in R^n$ are the estimated system states ($\hat{x}(t_j|\hat{\theta})$ represents the estimated variable at time t_j with parameter $\hat{\theta}$ and initial condition $\hat{x}_0$), W_i is the weighting matrix, $\hat{y}$ is the estimated measured data. In some applications, additional constraints are introduced to impose special structural properties of a given system; it can be implemented in the form of the equality and inequality constraints C_{eq} and C_{ineq} (for instance the system performance and the mass balance constraints). Finally, θ_L and θ_U are simple structural constraints such as the parameter's upper/lower bounds (it can be part of the C_{ineq}).

The basic idea of single shooting method is to estimate a set of parameters $\hat{\theta}$ and the initial condition $\hat{x}_0$. Then, putting $\hat{\theta}$ and $\hat{x}_0$ into the ODEs model and the estimated system states $\hat{x}$ can be computed. Also the error between the estimated system states $\hat{x}$ and the measurement data $y(t)$ can be expressed by (shown as Fig. S3)

$$e(t_j) = y(t_j) - \hat{y}(t_j|\hat{\theta}) = y(t_j) - g(\hat{x}(t_j|\hat{\theta})) \quad , \tag{3}$$

and we have the following cost function:

$$C(\hat{\theta}) = \sum_{j=0}^{N-1} (y(t_j) - \hat{y}(t_j|\hat{\theta}))^T W_j (y(t_j) - \hat{y}(t_j|\hat{\theta})) \tag{4}$$

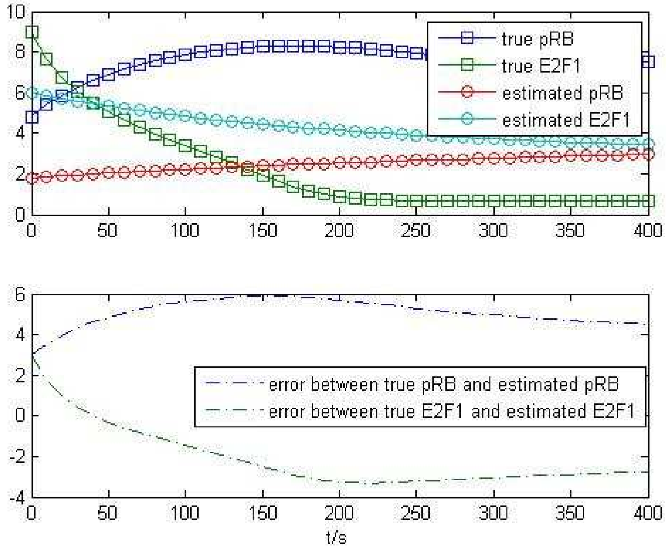

Fig. S3 True dynamic profile and the estimated dynamic profile

In order to simplify the analysis for exposition purpose, we only vary parameters ϕ_{pRB} and ϕ_{E2F1} over the ranges $\phi_{pRB} \in [0 \quad 0.025]$ and $\phi_{E2F1} \in [0 \quad 0.5]$ respectively, and fixed all other parameters at their nominal values. The following displays the cost function surface of P_0 .

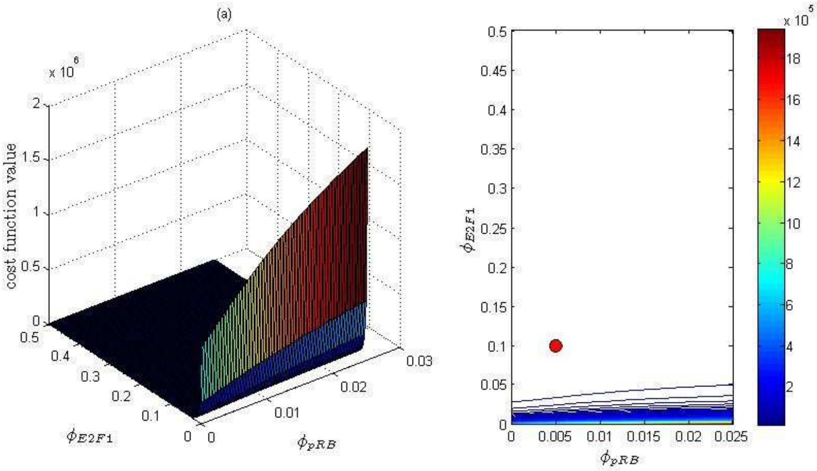

Fig. S4. Cost function surface of the P_0 as parameters ϕ_{pRB} and ϕ_{E2F1} are varied; (a) displays the cost function surface of P_0 , (b) the corresponding contour plots.

Fig. S4-(a) shows that the cost function surface of P_0 is ridge shape, which drops suddenly from 10^6 to 0 (the red dot, in Fig. S4-(b), is the minimal point). For the NLP- P_0 , the disadvantages of the single shooting method are: (i) single shooting method requires solving the nonlinear dynamic model (1) for $\hat{x}$ in order to compute the cost function. The common method to estimate $\hat{x}(t_i)$ and $\hat{x}(t_i)$ is using ODEs solvers, which perform the

numerical integration with $\hat{\theta}$ fixed at each iteration. During the process of identification, the integration has to be executed thousands even millions of times. Hence, the computation time spent on the P_0 can be hours even days. (ii) P_0 is a nonlinear optimization problem subject to a set of linear and non-linear differential equation constraints. Hence, P_0 is often multimodal (non-convex) and has many local minima. In a high-noise environment and combined with the non-convex property, the situation becomes more difficult. (iii) The estimated state may diverge away from the true trajectory, in some cases it may cause numerical overflow[1]. On the contrary, the single shooting method is simple in programming and there is no need to measure all states of the system.

S2. Decomposition method [2]

Single shooting method took a much longer time frame (horizon) to compute the estimated trajectory and hence requires more iteration for the estimation to converge to its true values. The computation time can be drastically reduced if one interprets the derivatives $\dot{x}$ at all measured time points t_k as slopes, estimates these slopes from the observation data $y(k_k)$. Then, the system model becomes:

$$\hat{\dot{x}}(t_j) = f(\hat{x}(t_j), u(t), \hat{\theta}) \tag{5}$$

If the metabolic profile consists of metabolites $\{x_1, x_2, ..., x_n\}$, which are measured at N instances $\{t_1, t_2, ..., t_N\}$,. The inverse problem can thus be reformulated from one involving n differential equations into a larger system of $n \times N$ algebraic equations as follows:

$$\begin{aligned} \hat{\dot{x}}_1(t_j) &= f(\hat{x}(t_j), u(t), \hat{\theta}_1) \\ \hat{\dot{x}}_2(t_j) &= f(\hat{x}(t_j), u(t), \hat{\theta}_2) \\ &\vdots \\ \hat{\dot{x}}_n(t_j) &= f(\hat{x}(t_j), u(t), \hat{\theta}_n) \end{aligned} \tag{6}$$

The error between the estimated system states $\hat{\dot{x}}_i(t_j)$ and its derivative based on the ODEs model $f(\hat{x}(t_j), u(t), \hat{\theta}_i)$ (shown as Fig. S5~S7) becomes

$$e_i(t_j) = \hat{\dot{x}}_i(t_j) - f(\hat{x}(t_j), u(t), \hat{\theta}_i) \tag{7}$$

Then, the parameter estimation problem can be separated into n small subproblems. Hence, the optimization function becomes

$$\begin{aligned} \mathrm{P}_{\mathrm{decoup,i}}:\ & \min_{\hat{\theta}_i} \sum_{j=0}^{N-1} \left\| \hat{\dot{x}}_i(t_j) - f(\hat{x}(t_j), \hat{\theta}_i) \right\|_2^2 \qquad (i = 1, 2, \cdots, n,) \\ s.t.\ & (i)\ \ \theta_L \le \hat{\theta} \le \theta_U, \end{aligned} \tag{8}$$

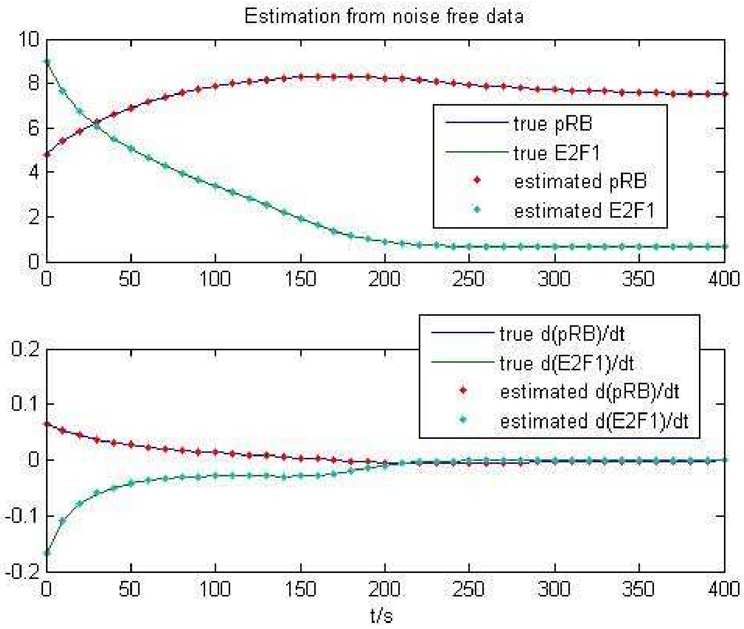

Fig. S5 observed dynamical profile and estimated dynamic profile in noise free condition

Then the cost function surface of (8) is shown in Fig. S6

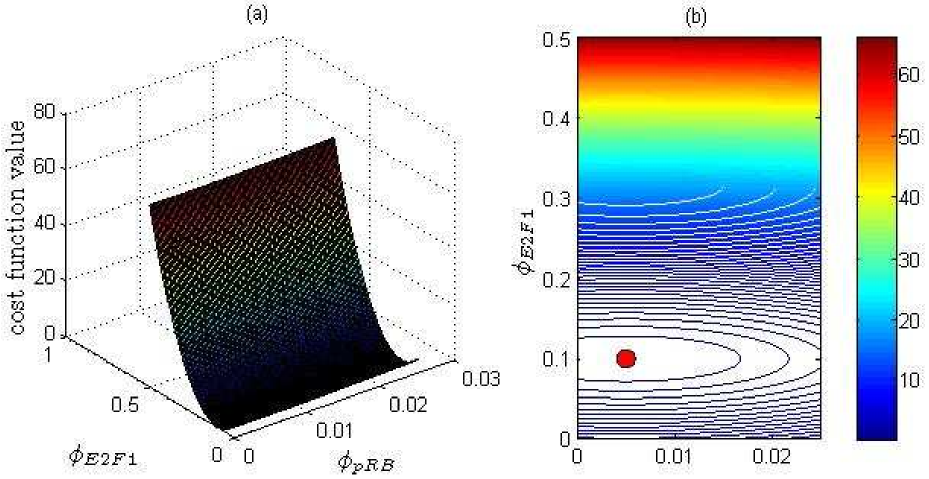

Fig. S6 (Color online); (a) Cost function surface of the P_{decoup} with parameters ϕ_{pRB} and ϕ_{E2F1} ; (b) corresponding contours of the cost function (red dot is the minimal point).

Compared with the cost function surface of P_0, the cost function surface of (8) is now bowl-shaped and local convex, which is "more smooth". However, the reliability of the decomposed methods depends on the accuracy of the estimated states $\hat{x}(t_j)$ and their derivative $\hat{\dot{x}}(t_j)$. In practice, these data are subject to significant observation noise. Without proper regularization pre-processing, the overfitting problem may occur (shown in Fig. S7).

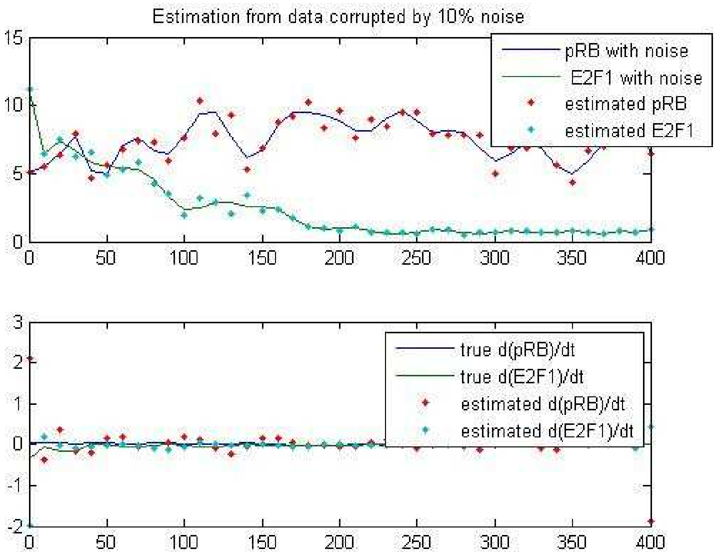

Fig. S7 The observed dynamical profile and estimated dynamic profile in 10% noise condition

Under this condition, the optimal solution can deviate from the "true" value [3]. One can see from Fig S8. The red point shown in Fig S8(b) deviates from the "true" value $\phi_{pRB} = 0.005$, $\phi_{E2F1} = 0.1$

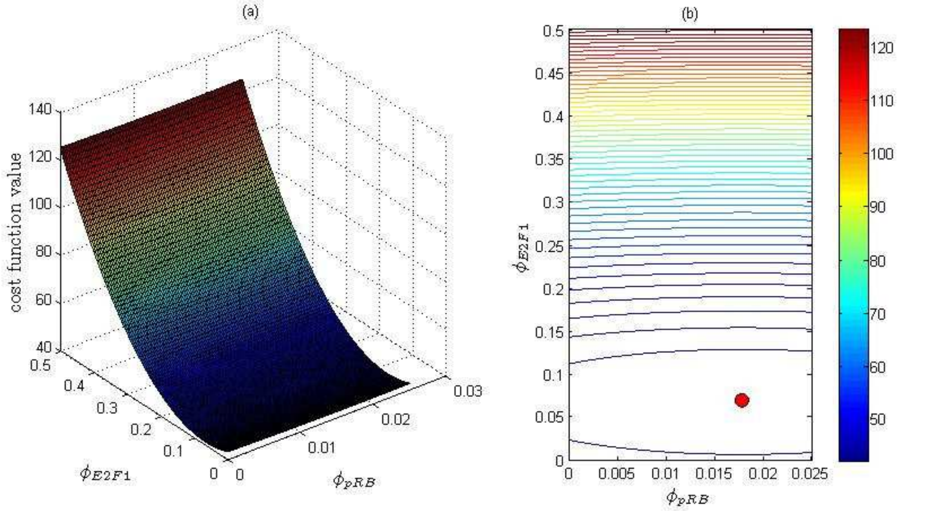

Fig. S8 (Color online); (a) Cost function surface of the P_{decoup} as parameters ϕ_{pRB} and ϕ_{E2F1} are varied; (b) corresponding contours of the cost function (red dot is the minimal point).

S3. Combine spline theory and NLP (The proposed method P_3)

The parameter estimation problem is given as follows

$$\mathrm{P}_3:\ \min_{\hat{\theta},p} \sum_{j=0}^{N-1} (y(t_j) - \hat{y}(t_j \,|\, \hat{\theta}))^T w_j (y(t_j) - \hat{y}(t_j \,|\, \hat{\theta}))$$

$$s.t.\ \begin{cases} \text{(i)}\ \ \hat{x}_i(t_j) = b_i^T(t_j)\cdot p_i, \\ \text{(ii)}\ \ \dot{\hat{x}}_i(t_j) = \dot{b}_i^T(t_j)\cdot p_i, \\ \text{(iii)}\ \ \left\| \dot{\hat{x}}(t_j) - f(\hat{x}(t_j), u(t), \hat{\theta}) \right\|_2^2 = 0, \\ \text{(iv)}\ \ \hat{y}(t_i) = g(\hat{x}(t_j)), \\ \text{(v)}\ \ \theta_L \le \hat{\theta} \le \theta_U, \\ \quad i = 1, 2, \cdots, n, \quad j = 0, 2, \cdots, N-1. \end{cases} \tag{9}$$

Now, the NLP- P_3 consists of algebraic equation constraints only. Hence, P_3 does not require ODEs solvers, which drastically ease the computation burden.

Then, we can turn P_3 into

$$\mathrm{P}_4:\ \min_{\hat{\theta},p} \sum_{j=0}^{N-1} (y(t_j) - g(B(t_j)\cdot p))^T w_j (y(t_j) - g(B(t_j)\cdot p))$$

$$s.t.\ \begin{cases} \text{(i)}\ \ \left\| \dot{B}\cdot p - f(B\cdot p, B_{\hat{\tau}}\cdot p, \hat{\theta}) \right\|_2^2 = 0, \\ \text{(ii)}\ \ \theta_L \le \hat{\theta} \le \theta_U, \quad i = 1, 2, \cdots, n, \quad j = 0, 2, \cdots, N-1. \end{cases} \tag{10}$$

where $p = (p_1 \quad p_2 \quad \cdots \quad p_L)^T$.

The Lagrangian function of P_4 is

$$L(\hat{\theta}, p, \lambda) = \sum_{j=0}^{N-1} (y(t_j) - B(t_j)\cdot p)^T w_j (y(t_j) - B(t_j)\cdot p) + \lambda \left\| \dot{B}(t_j)\cdot p - f(B(t_j)\cdot p, B_{\hat{\tau}}(t_j)\cdot p, \hat{\theta}) \right\|_2^2, \tag{11}$$

where $\lambda > 0$ is the Lagrange multiplier associated with the constraint (i) of (10). In conclusion, the parameter estimation problem becomes a minimization problem as follows:

$$\mathrm{P}_4:\ \min_{\hat{\theta},p} \left(\sum_{j=0}^{N-1} (y(t_j) - B\cdot p)^T w_j (y(t_j) - B\cdot p) + \lambda \left\| \dot{B}\cdot p - f(B\cdot p, \hat{\theta}) \right\|_2^2 \right) \tag{12}$$

over the search range $\theta_L \le \hat{\theta} \le \theta_U$. Compared with the decomposed methods which divide the estimation of the system states (and its derivative) and the parameter estimation into two separate steps; while P_4 computes the estimated states (and its derivative) and parameters at the same time. Note that constraint (iii) of P_3 governs the estimation of system's states (and their derivative) so as to make sure these estimates belong to one solution of system (1). Thus, the dynamic system model itself servers as a filter performing regularization and the overfitting problem can be relieved Hence, even in noisy condition, the position of minimal point do not derivate as badly as the decomposition method (shown as Fig S8 and S9).

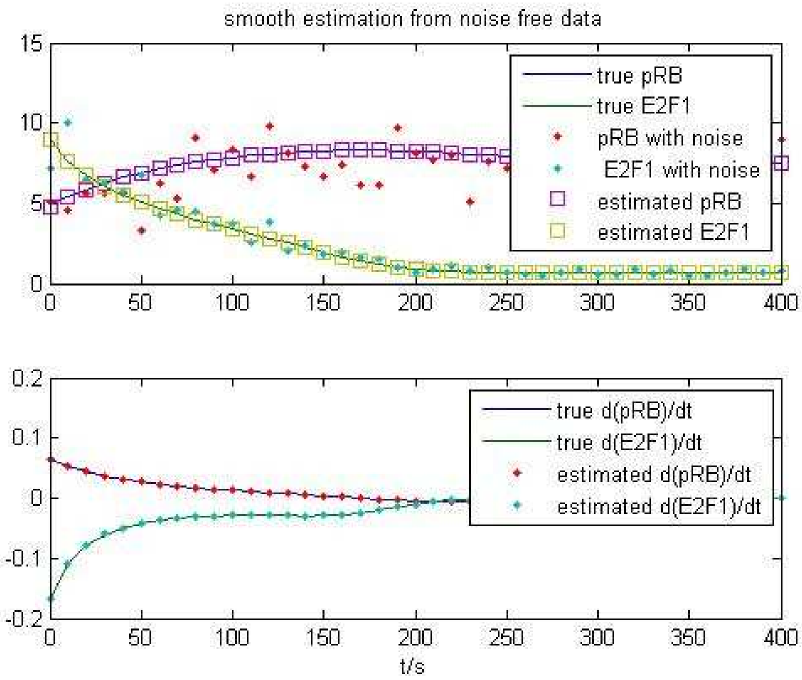

Fig. S8 observed dynamical profile and estimated dynamic profile in 10% noise condition

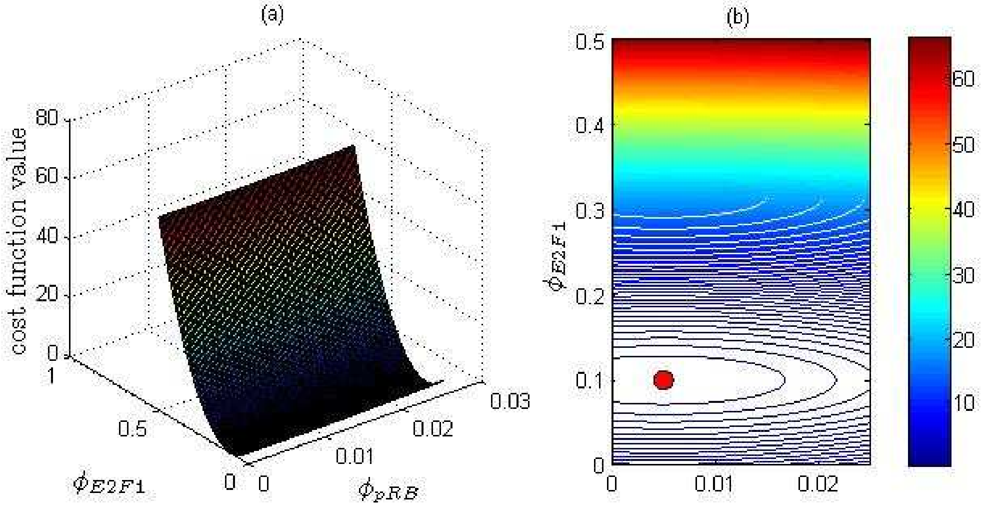

Fig. S9 (Color online); (a) Cost function surface of the P_{decoup} as parameters ϕ_{pRB} and ϕ_{E2F1} are varied are varied; (b) corresponding contours of the cost function (red dot is the minimal point).

Hence, the augmented cost function surface of P_3 is smooth. This characteristic enables the optimization algorithm to arrive at the best solution more easily.

Reference

[1] M. Peifer and J. Timmer, “Parameter estimation in ordinary differential equations for biochemical processes using the method of multiple shooting”, IET Syst. Biol., 2007, 1, (2), pp. 78-88.
[2] Voit EO, Almeida J. (2004) “Decoupling dynamical systems for pathway identification from metabolic profiles”, Bioinformatics. 20(11):1670-81.
[3] Marco Viela, I-Chun Chou, Susana Vinga, Ana Tereza R Vasconcelos, Eberhard O Voit and Jonas S Aleida, “Parameter optimization in S-system models”, BMC Systems Biology, 2008, 2:35.
[4] Swat M, Kel A, Herzel H. (2004) “Bifurcation analysis of the regulatory modules of the mammalian G_1/S transition”, Bioinformatics. 20(10):1506-1511.
